# Exploring pharmaphylogeny from multiple perspectives: a case study on Lithospermeae

**DOI:** 10.1038/s41598-023-34830-4

**Published:** 2023-05-11

**Authors:** Yumei Yan, Xinxin Wei, Bin Qiu, Guoping Wang, Baochang Zhou, Mingxu Zhang, Yibo Liu, Siqi Li, Bowen Gao, Minhui Li

**Affiliations:** 1 Department of Pharmacy, Baotou Medical College, Baotou, 014040 China; 2 Pharmaceutical Laboratory, Inner Mongolia Hospital of Traditional Chinese Medicine, Hohhot, 010000 China; 3 Department College of Life Sciences, Inner Mongolia University, Hohhot, 010000 China; 4 Department of Traditional Chinese Medicine, Yunnan University of Chinese Medicine, Kunming, 650000 China; 5 Xinjiang Key Laboratory of Chinese Materia Medica and Ethnic Materia Medica, Xinjiang Institute of Chinese Materia Medica and Ethnical Materia, Xinjiang, 830000 China; 6 Department of Traditional Chinese Medicine, Inner Mongolia Medical University, Hohhot, 010000 China

**Keywords:** Plant sciences, Plant evolution

## Abstract

Lithospermeae Dumort., a tribe under the subfamily Boraginoidae, is a perennial herb containing approximately 470 species under 26 genera, primarily distributed in temperate and tropical regions. To gain a deeper understanding of the medicinal plants of Lithospermeae and better protect and develop plant medicinal resources, the phytochemistry, pharmacology, and traditional use of Lithospermeae with medicinal value were analyzed. Phylogenetic analysis was carried out based on the internal transcribed spacer sequence. Through spatial analysis and the species distribution model, the spatial distribution pattern of Lithospermeae medicinal plants was analyzed. Meanwhile, the relevant targets and pathways involved in the pharmacological effects of commonly used medicinal plants were predicted using network pharmacology to further explore the genetic origin of Lithospermeae and enrich the pharmaphylogeny of medicinal plants. In this study, the chemical composition, traditional efficacy, and modern pharmacological activity of Lithospermeae were collected for the first time and analyzed in combination with the geographical distribution model, molecular phylogeny, and network pharmacology. Based on our findings, the pharmaphylogeny of Lithospermeae was preliminarily discussed, providing the scientific basis for basic research regarding Lithospermeae. Concurrently, this study explored the relationship between the development of the regional medicinal plant industry and the protection of biodiversity. Furthermore, our findings provide direction and theoretical guidance for the study of the phylogenetic relationships in medicinal plants and the development of Lithospermeae medicinal plant resources.

## Introduction

Pharmaphylogeny is a frontier subject that studies the relationship among medicinal plant genetics, chemical composition, and efficacy, which includes both pharmacological activity and traditional efficacy. Pharmaphylogeny as a field is interdisciplinary, and the research objects involve multidisciplinary fields, including plant phylogeny, plant taxonomy, plant chemistry, pharmacology, molecular systematics, genomics, etc. Plants with similar genetic relationships have been emphasized to contain similar chemical or bioactive components, as well as similar therapeutic effects^1,2^. In the early stage of the development of pharmaphylogeny, the cladistic systematics method primarily focused on morphological characteristics that relied on the previous work of species identification and character differentiation. However, the error concurred using this approach was large. In recent years, with the development of molecular biology, the relationship between plant morphology and molecular systematics has become the main approach used to study the systematic relationship between plants^3,4^. Species under the same genus have been observed to have similar morphological characteristics and no significant differences in molecular sequences, indicating that they are closely related to each other. Concurrently, radiation evolution widely exists in plants^5^. The adaptive radiation and isolated differentiation of species under the same genus may originate from climate change, serving as the main driving force for radiation evolution in many plant lineages^6^. As such, climate change has the potential to lead to variations in the same species, including changes in chemical composition and biological activity^7^. Therefore, we introduce geographical distribution as a useful supplement to the pharmaphylogeny theory. Lithospermeae has a wide distribution of species and a rich biodiversity. Notably, the species distribution data obtained in this study were different from those previously reported. As researchers provide more data in the Global Biodiversity Information Facility (GBIF, https://www.gbif.org/ ) database, species distribution results can be enriched and improved. Through this, we hope to explore the association between climate and genetic relationships among species.

Lithospermeae, a tribe of Boraginoideae, includes 26 genera and approximately 470 species^8^. Lithospermeae exhibit several characteristics typical of Boraginaceae s.str., including a predominantly herbaceous habit, the characteristically bristly indument of stiff trichomes, the sympetalous corolla, commonly in colors of corolla yellow, white, or bluish purple, and the fruit consisting of four nutlets. *Alkanna* Tausch., *Arnebia* Forssk., *Echium* L., *Lithospermum* L., *Lobostemon* Lehm., *Onosma* L., and *Stenosolenium* Turcz., constitute a closely related genera group of Lithospermeae with very similar plant morphology^8–15^ (http://www.iplant.cn/). Lithospermeae are widely distributed in temperate and tropical regions around the world^8^. Lithospermeae primarily includes *Arnebia*, *Echium*, *Lithospermum*, *Onosma*, *Stenosolenium*, and others^8,16^. The roots of Lithospermeae medicinal plants were traditionally used for fever palliation, scald burn relief, detoxification, detumescence, pain relief, indigestion relief, etc.^17^. Lithospermeae plants contain an abundance of chemical constituents, including naphthoquinone, flavonoids, phenolic acids, and other bioactive substances^18–22^. Modern pharmacological studies have demonstrated that Lithospermeae plants generally have anti-inflammatory, antioxidant, antibacterial, antiviral, and antitumor activities^17,23–25^. Due to the rich biological activities of Lithospermeae, it has gradually attracted people's attention and become a hot topic of research. However, the relationship among genera in Lithospermeae remains unclear. Moreover, most species have not yet been developed as medicinal plants and limits the development and utilization of Lithospermeae^2^.

In this study, the pharmaphylogeny study of Lithospermeae plant groups was performed in combination with molecular phylogeny, geographical distribution, chemical composition, traditional applications, and pharmacological effects. Moreover, we used network pharmacology to predict the potential molecular correlation between the chemical composition of Lithospermeae medicinal plants and its anti-inflammatory, antitumor, and anti-anxiety effects. The study of the pharmaphylogeny of Lithospermeae from multiple fields and disciplines provides novel ideas for the development of pharmaphylogeny and scientific evidence for the development of medicinal plants.

## Results

### Plant systematics of Lithospermeae

**Figure 1:**
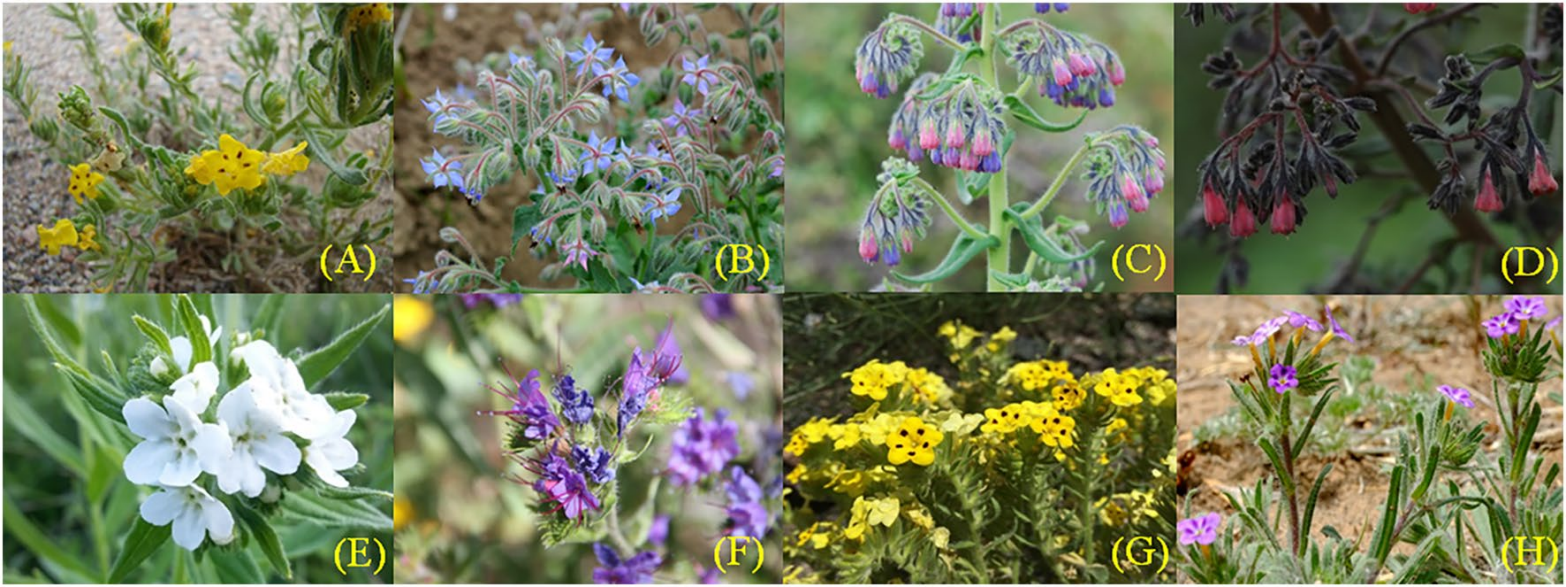
Morphology of some medicinal plants of Lithospermeae. (**A**: *Arnebia decumbens* (Vent.) Coss. et Kral., **B**: *Onosma exsertum* Hemsl., **C**: *Onosma confertum* W. W. Smith., **D**: *Onosma paniculatum* Bur. et Franch., **E**: *Lithospermum erythrorhizon* Sieb. et Zucc., **F**: *Echium vulgare* L., **G**: *Arnebia guttata* Bge., H: *Stenosolenium saxatile* (Pallas) Turcz.).

In the phylogenetic tree, there are mainly four branches, with the first branch including *Onosma* and *Maharanga* DC. There are main notable morphological differences between *Maharanga* and *Onosma*. *Maharanga* has a ovoid-ellipsoid or obovoid corolla, contracted at both ends, abruptly expanding from a short tube into a relatively large inflated throat, with inflated ribs below lobes and deep furrows between them; throat unappendaged. In contrast, *Onosma* has a simple bell-shaped corolla, corolla blue, yellow, rarely white or red, tubular-campanulate or retrorse-conical, usually gradually expanded from the base upward, rarely abruptly expanded from the middle; throat unappendaged. Additionally, the base and the side of its anther are united^26^. Pollen morphology is a conservative characteristic of *Onosma*^26^. Figure 1 illustrates the morphology of some medicinal plants of Lithospermeae.

The second branch consists of *Echium*, *Lobostemon*, and *Echium* and begins as a cluster of panicles that gradually unites. It is obliquely funnel-shaped and is pubescent outside. The basal and lower leaves are blade lanceolate, the base is gradually narrowed into a handle, and long, rough hairs are present on both sides. Middle leaves. It is a corolla usually purplish-blue. There are two different classifications of *Echium*, viewing it either as an independent tribe of Boraginoideae or an entity under Lithospermeae. Evolution-wise, the pollen of *Echium* is at a higher level than the tricolpate pollen of *Onosma*. Based on the types of pollen pores, the authors believe that *Echium* has a classic tricolpate pollen, supporting the classification of *Echium* under Lithospermeae^27^. *Lobostemon* is characterized via a dry, papillary bilobed stigma and a hairless appearance^4^.

**Figure 2:**
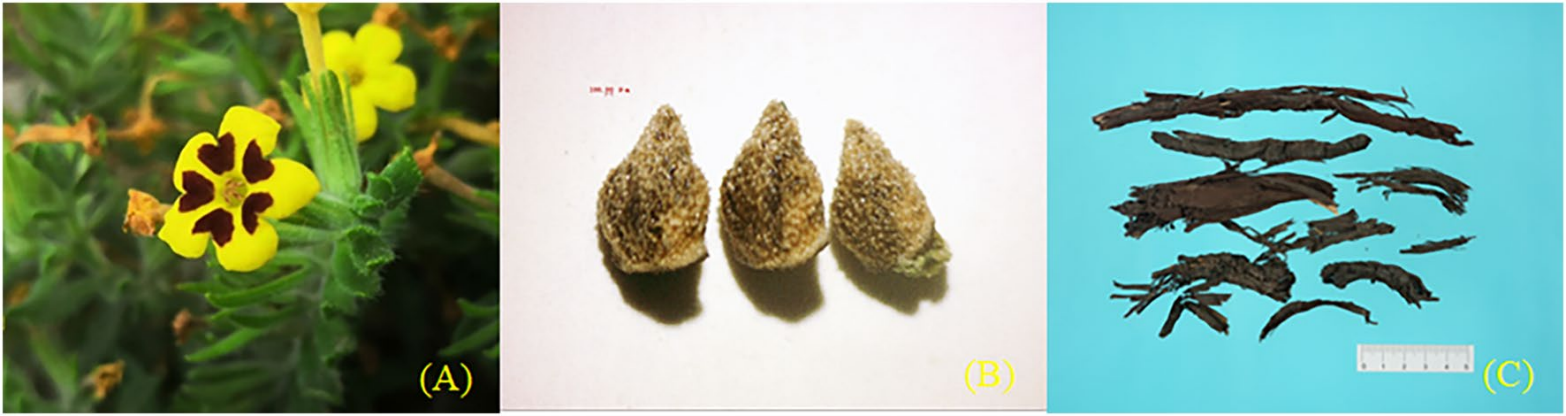
The pictures related to morphology and specific plant parts of *Arnebia guttata* Bge (**A**. Corolla **B**. Seeds **C**. Dried root (main medicinal parts)).

**Figure 3:**
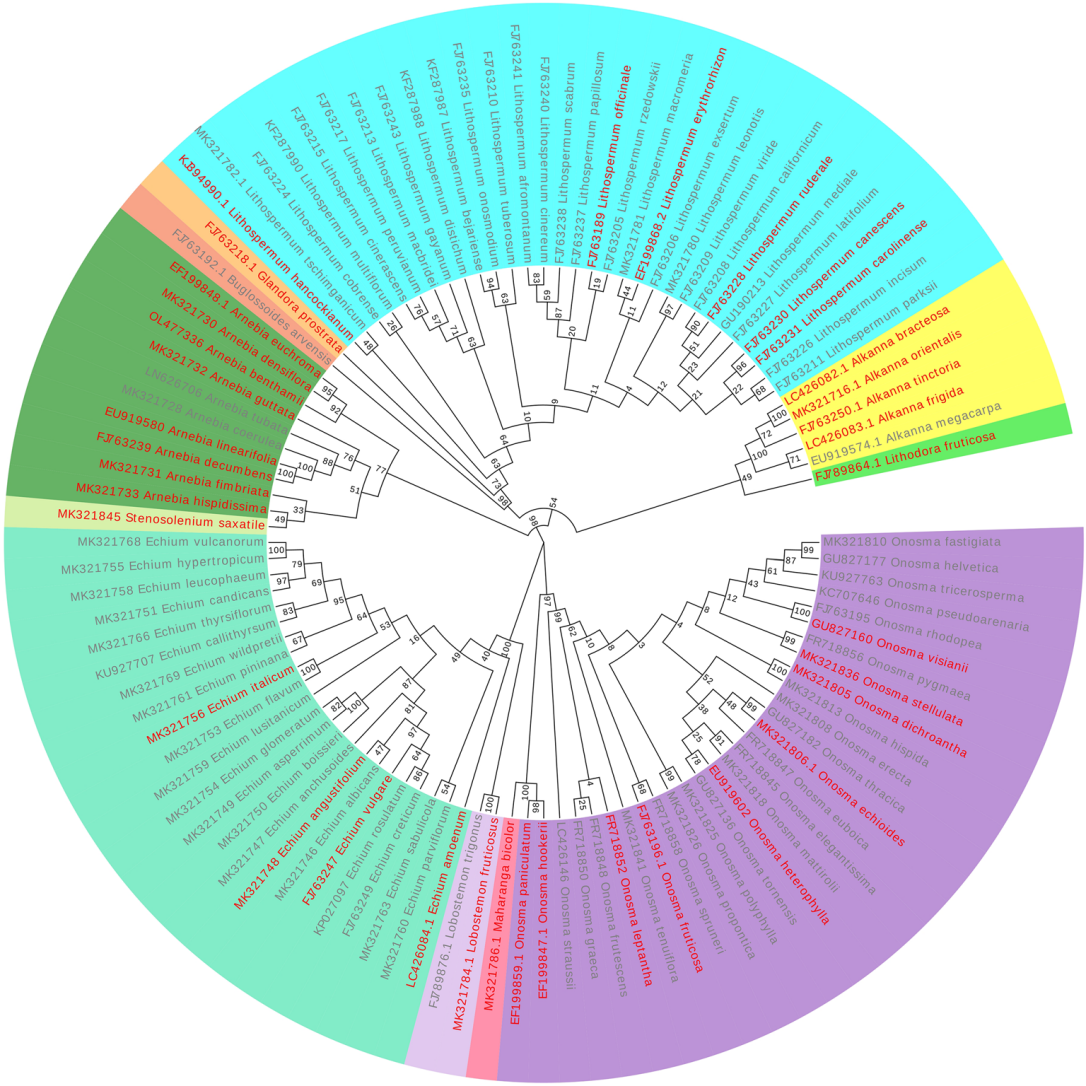
Phylogenetic tree of Lithospermeae species (The red font is a medicinal plant of Lithospermeae).

The third branch consists of *Alkanna*, *Arnebia*, *Buglossoides* Moench, *Glandora* D.C.Thomas, Weigend & Hilger, *Lithodora* Griseb, *Lithospermum*, and *Stenosolenium*. *Alkanna* has basal leaves oblanceolate, eglandular white-tomentose with a dense indumentum of very short crisped hairs, and a yellow corolla. *Lithodora* has apparently stylar polymorphic since populations of all these species were found to have two morphs for style length: one morph with styles above the anthers (approach herkogamous) and the other positioned below (reverse herkogamous). Flowers are actinomorphic and sympetalous, form a floral tube, and have five small stamens inserted in the corolla tube. *Lithospermum* has short strigose, corolla actinomorphic, funnelform or salverform, throat with appendages or bands of hairs, or longitudinally crispate. Nutlets white or gray, ovoid, smooth, shiny or tuberculate; attachment scar at base adaxially. *Buglossoides* inflorescences are basically frondose-bracteose cincinni (“cymoids”) with two to several flowers, usually distinctly elongating in fruit and with well-spaced, nutlet-bearing calyces. Its corolla may appear discoid and are in colors of white, blue, or light blue. *Buglossoides* is a small genus originally based on *Lithospermum tenuiflorum* and comprises seven species native to the Old World, mostly concentrated in the Mediterranean region. *Glandora* is a perennial shrub with flowers on top of leafy branches. Flowers are linear and petal-shaped, forming tubes. There are five small stamens in the corolla tube. *Arnebia* is an annual or perennial herb with bristly or pubescent. Flowers are often heterostylous. Corolla funnelform, commonly with hairs outside. Nutlets oblique-ovate, tuberculate, adaxially flat or slightly concave. The roots contain purple substances. (Fig. 2) Finally, *Stenosolenium* is a perennial herb with slender roots. Leaves are oblanceolate or lanceolate. Its corolla may appear purple, cyanish purple, or white. It has a slender tube and a hairy ring base. Figure 3 illustrates the phylogenetic tree of Lithospermeae species.

### Geographical distribution

**Figure 4:**
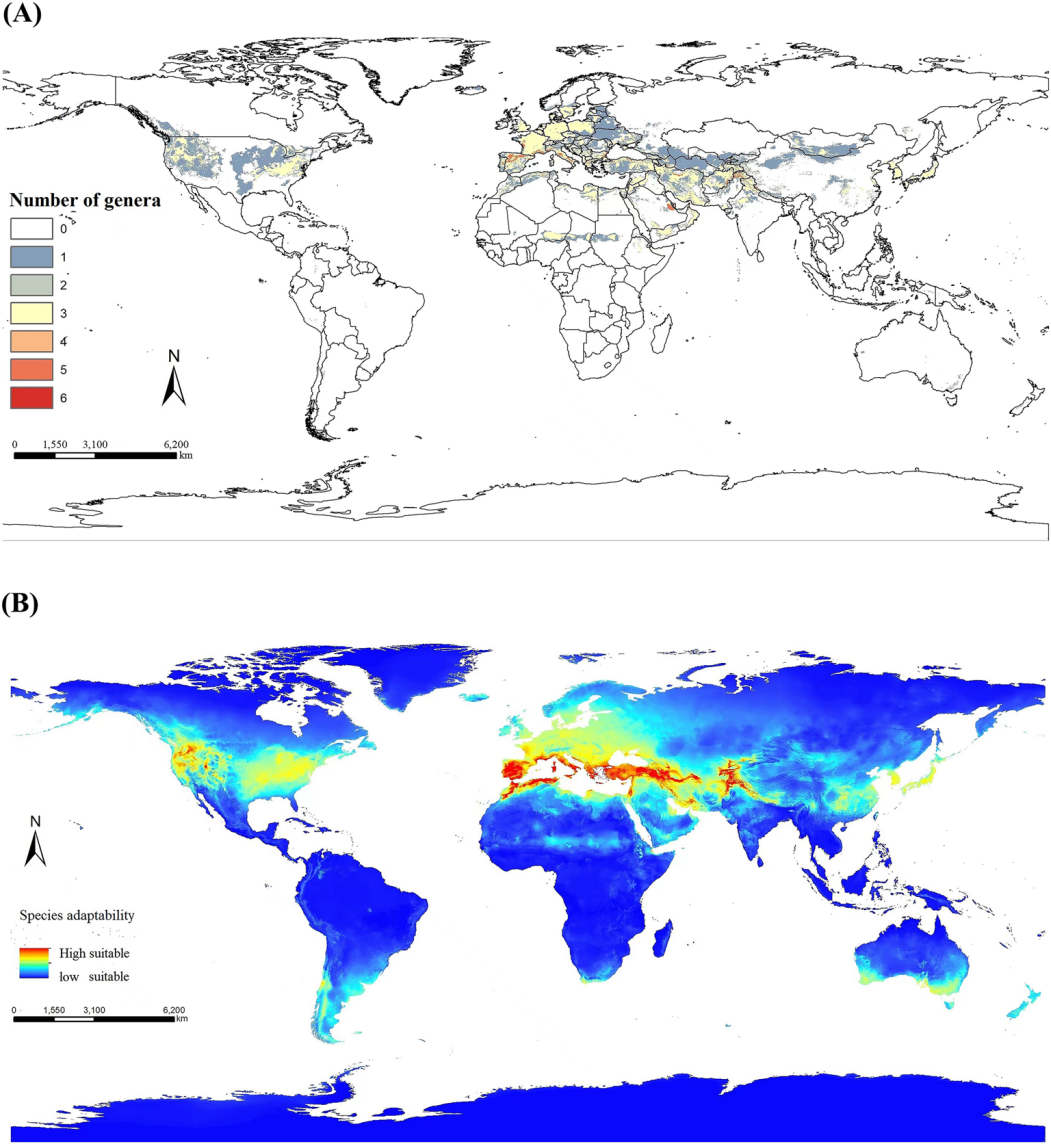
(**A**) Genera of medicinal plants of Lithospermeae, the overlapping of the main distribution areas is at least 0 and at most 6 genera. (**B**) Species adaptability distribution map of Lithospermeae.

The medicinal plants of Lithospermeae are mainly distributed between 61° 14′ N and 14° 35′ N. Except for those in tropical rainforests, cold climates, tropical deserts, other climatic areas have distribution and abundant resources. The spatial distribution pattern illustrates high diversity in the Mediterranean climate regions, mainly including the Mediterranean coast, the Black Sea coast, southern Europe, and the northern African coast. High diversity is also observed in a few areas in western Asia, the western coast of southern Africa, and southwest and southeast coasts of Australia. The climate in these regions is usually hot and dry during the summer, and rainy in the winter. Notably, Lithospermeae species diversity is decreased at low latitudes. *Alkanna* is primarily distributed along the Mediterranean coast and the western coast of North America. *Echium* is mainly distributed along the Mediterranean coast and the western region of North America. *Arnebia* is primarily distributed in Africa and Asia. *Lithospermum* is mainly distributed in Asia. *Onosma* is mainly distributed along the Mediterranean coast and Tibet in China. *Lithodora* is primarily distributed near the Mediterranean Sea. *Lobostemon* is located in the southwest corner of Australia. *Stenosolenium* is distributed in eastern Asia. Figure 4A shows the distribution range of Lithospermeae. Moreover, the main distribution map of each genus is shown in the attachment (Supplementary Fig. S1–8).

From the perspective of suitable ecological zones, *Alkanna* is suitable for growing along the Mediterranean coast, central Asia, and western North America. *Echium* is suitable for growing along the Mediterranean coast, North America, southern South America, southwest Australia, and southeast Australia. *Arnebia* is suitable for growing in northern Africa and central Asia, along the Mediterranean coast. *Lithospermum* is suitable in eastern Asia and the Mediterranean coast, Europe, and North America. *Onosma* is more suitable on the Mediterranean coast. *Lithodora* is suitable for growing along the Mediterranean coast, along the western coast of North America, and the southeast corner of Australia. *Lobostemon* is suitable to grow in North America, Europe, and eastern Asia. *Stenosolenium* is suitable for growing in eastern Asia and parts of North America. Figure 4B shows the suitability distribution of Lithospermeae medicinal plants and the distribution trend of each genus is shown in the attachment. (Supplementary Fig. S9–17).

### Chemical constituents

Research on the plant components of Lithospermeae mainly focuses on *Arnebia*, *Echium*, *Lithospermum*, and *Onosma*, followed by *Alkanna*, *Lobostemon*, and *Stenosolenium*. and *Buglossoides*, *Lithodora*, and *Maharanga* have been rarely reported^30,41,44^. The core components of Lithospermeae are purple naphthoquinones (e.g., shikonins, alkannins), pyrrolizidine alkaloids, and phenolic acids. The distribution of chemical constituents in each genus is summarized in Table 1. (The chemical structures file is attached. Supplementary Table S1).

**Table 1:** Distribution of Chemical Species in Lithospermeae (Notes: + represents 1–5 compounds; others of quinones: benzene quinones, anthraquinones etc., others of phenolic acids:caffeic acid, ferulic acid etc., Others: terpenoids etc.).

Genus	Quinones					Phenolic acids				Pyrrolizidine alkaloids	Flavonoids	Aliphatic and ester compounds	Others
	Shikonins	Alkannans	shikonin dimer	Furans	Others	Caffeic acid dimer	Caffeic acid trimer	Caffeic acid tetramers	Others				
*Alkanna*	+	++			+	+				+	+		
*Arnebia*	+++++	+++++	+	+	+++++	+		+	+	++	+	+	+++
*Buglossoides*												+	
*Echium*	+++	+				+			++	+++	+	+	++
*Glandora*						+	++	+	+		++	+++	++
*Lithodora*												+	
*Lithospermum*	++++	++	+	++++	+	+	+		+	++	+		++
*Lobostemon*						+	+		+	+	+ +	+	+
*Maharanga*		+											
*Onosma*	+ + +	+ +		+	+	+			+ +	+ + +	+ +	+	
*Stenosolenium*	+	+				+	+		+	+			

### Traditional application and associated pharmacological activity

Traditionally, Lithospermeae medicinal plants are primarily used for burns, diarrhea, abscess, fever, detoxification, swelling, promoting blood circulation, dispelling wind pain, and contraception. Clinically, they are mainly used for their anti-inflammatory, antibacterial, antitumor, antioxidant, and wound-healing effects. The summary of its traditional application and pharmacological effects in individual genus distribution is shown in Table 2.

**Table 2:** Traditional applications and biological activities of Lithospermeae plants.

Genus	Traditional applications	Biological activity	References
*Alkanna*	Diarrhea Abscess Wounds Burns Sore throat	Analgesia Antibacterial Anticancer Antimicrobial Anti-inflammatory Anti-nociceptive Antioxidant Antiviral Cutaneous leishmaniasis Wound healing Antipyretic Sedative effects	^17,45–55^
*Arnebia*	Antipyretic Bactericidal Boils Burns Clearing heat and detoxifying Colic pain Cooling blood Detoxify Detumescence Fever Headache Heart disease Helminthiasis Promoting blood circulation Rash Skin diseases Stomach pain Wounds	Anti-aids Antibacterial Antibiotic Anticancer Antidermatophytic Antifungal Anti-inflammatory Antimicrobial Antioxidant Antithrombotic Antitumor Antiviral Wound healing Anti-hcv	^17,56–66^
*Buglossoides*	Weight loss	Antioxidant	^17,41^
*Echium*	Abscess Antianxiety Contusion Cooling blood Diuresis Diuretic Expectorant Hand crack Lubricant Protective agent Rheumatic pain Sedation Trauma Trauma	Acute and chronic constipation Analgesic Antianxiety Antibacterial Anticonvulsant Antifungal Anti-inflammatory Antimicrobial Antioxidant Antitumor Antiviral Anxiolytic Delayaging Hemostasis Hypolipidemia Insecticidal Lower blood pressure Prevention of alzheimer's disease Wound healing Demulcent	^17,67–78^
*Glandora*	Diuretic Depurative Anti-hypertensive	Anticancer	^100,101^
*Lithodora*	Blood purification Chilblain Cold Cough Enteritis Fever Flu Hypertension Liver protection Pneumonia Rheumatism Sedation Stomachache		^17^
*Lithospermum*	Analgesia Bronchitis Clearing heat and detoxifying Contraception Cooling blood Cough Detumescence Diminishing inflammation Dispelling wind Diuretics Dyspepsia Fever Fracture Gastric distention Hematemesis Hemostasis Joint pain Promoting blood circulation Rash Relieving pain Sore Swelling Traumatic Weakness	Angiostatic Antibacterial Anticancer Antifertility Antigonadotropic Antihormonal Anti-inflammatory Antimicrobial Antioxidant Antithrombotic Antitumor Antiviral Immunostimulatory Lymphatic tuberculosis Pulmonary tuberculosis Wound healing	^17,40,79–89^
*Lobostemon*	Bacteriosis Ulcer Wounds	Anti-aids Wound healing	^17,24,37^
*Maharanga*	Constipation	Antibacterial Antiviral	^17,30^
*Onosma*	Abdominal Alexipharmic eye diseases Anthelmintic Blood disorders Bronchitis Burns Clearing heat and detoxifying Cooling blood Foot ulcers Heart disease Itch Laxative Lung cancer Pain Promoting blood circulation Rash Respiratory diseases Rheumatism	Antibacterial Anticancer Antifungal Anti-inflammatory Antimicrobial Antioxidant Antiproliferativie Antithrombosis Antitumor Hypoglycemic Respiratory diseases Tetranychusurticae Wound healing Antidiabetic	^17,90–99^
*Stenosolenium*	Clearing heat Cold Cooling blood Cough Dehumidification Dispelling wind Hematemesis Joint pain Lung heat Respiratory diseases Stopping bleeding		^17^

### Network pharmacology study on representative genera of Lithospermeae

#### Screening of the active components of *Lithospermum*, *Arnebia*, and *Echium* and subsequent prediction of their targets

Through drug-likeness (DL) and high gastrointestinal absorption (GI) screening conditions, 25 active components of *Lithospermum*, 26 active components of *Arnebia*, and 16 active components of *Echium* were obtained (Table 3). Furthermore, 571 component targets of *Lithospermum*, 549 component targets of *Arnebia*, and 399 component targets of *Echium* were predicted by the Swiss Target Prediction database (http://swisstargetprediction.ch/).

**Table 3:** *Arnebia*, *Echium* and *Lithospermum* screened active compounds (Notes: GI absorption: gastrointestinal absorption; DL: drug likeness).

Compounds	GI absorption	DL	Source
1,8-Cineole	High	3yes	*Arnebia*, *Lithospermum*
2-methyl-n-butyryl shikonin	High	5yes	*Echium*
4-hydroxybenzoic acid	High	3yes	*Echium*
7-tigloylretronecine	High	5yes	*Arnebia*, *Lithospermum*
acetylshikonin	High	5yes	*Echium*, *Lithospermum*
alkannan	High	5yes	*Echium*
angelylalkannin	High	5yes	*Arnebia*, *Lithospermum*
anhydroalkannin	High	5yes	*Arnebia*, *Lithospermum*
arnebifuranone	High	5yes	*Arnebia*, *Lithospermum*
arnebinone	High	5yes	*Arnebia*, *Lithospermum*
caffeic acid	High	4yes	*Echium*
cyanidin	High	5yes	*Echium*
deoxyalkannin	High	5yes	*Arnebia*, *Lithospermum*
deoxyshikonin	High	5yes	*Arnebia*
echimidine	High	5yes	*Arnebia*, *Lithospermum*
geranylbutyrate	High	5yes	*Arnebia*, *Lithospermum*
glabridin	High	5yes	*Arnebia*, *Lithospermum*
hydrocaffeic acid	High	4yes	*Echium*
isobutyrylshikonin	High	5yes	*Arnebia*, *Echium*, *Lithospermum*
isovalerylalkannin	High	5yes	*Arnebia*, *Lithospermum*
isovalerylshikonin	High	5yes	*Arnebia*, *Echium*, *Lithospermum*
jolkinolide E	High	5yes	*Arnebia*, *Lithospermum*
leptanthine	High	4yes	*Echium*
lycopsamine	High	4yes	*Arnebia*, *Lithospermum*
osthol	High	5yes	*Arnebia*, *Lithospermum*
p-coumaric acid	High	4yes	*Echium*
p-hydroxybenzoic acid	High	3yes	*Echium*
pinoresinol	High	5yes	*Echium*
propionylalkannin	High	5yes	*Arnebia*, *Lithospermum*
propionylshikonin	High	5yes	*Arnebia*, *Echium*, *Lithospermum*
shikonofuran A	High	5yes	*Arnebia*, *Lithospermum*
shikonofuran E	High	4yes	*Arnebia*, *Lithospermum*
supinine	High	5yes	*Arnebia*, *Lithospermum*
syringic acid	High	4 yes	*Echium*
teracrylshikonin	High	4yes	*Arnebia*, *Lithospermum*
vanillic acid	High	4yes	*Arnebia*, *Echium*, *Lithospermum*
*β*-hydroxyisovalerate	High	3yes	*Arnebia*
*β*-Santalol	High	4yes	*Arnebia*, *Lithospermum*

#### Obtaining disease targets

In Online Mendelian Inheritance in Man (OMIM) and GeneCards databases, disease targets were searched using the keywords “cancer”, “inflammation”, and “anxiety disorder”. Targets with scores ≥ 5 were selected as potential targets. After merging the results of the two databases and deleting duplicates, 2976 cancer targets, 569 inflammatory targets, and 2102 anxiety disorder targets were obtained. Using the Venny 2.1.0 online platform, the intersection of component targets and disease targets was obtained. A total of 247 intersecting targets of *Lithospermum* and cancer, 98 intersecting targets of *Arnebia* and inflammation, and 156 intersecting targets of *Echium* and anxiety disorder were obtained.

### KEGG pathway analysis and construction of “active ingredient-target-pathway” network

**Figure 5:**
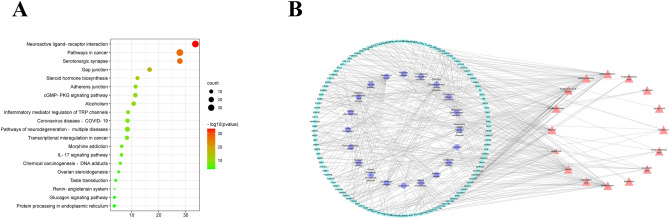
(**A**) Analysis of the anti-anxiety KEGG pathway in *Echium*; (**B**) Anti-anxiety Chemical Composition—Target—Pathway Network of* Echium.*

**Figure 6:**
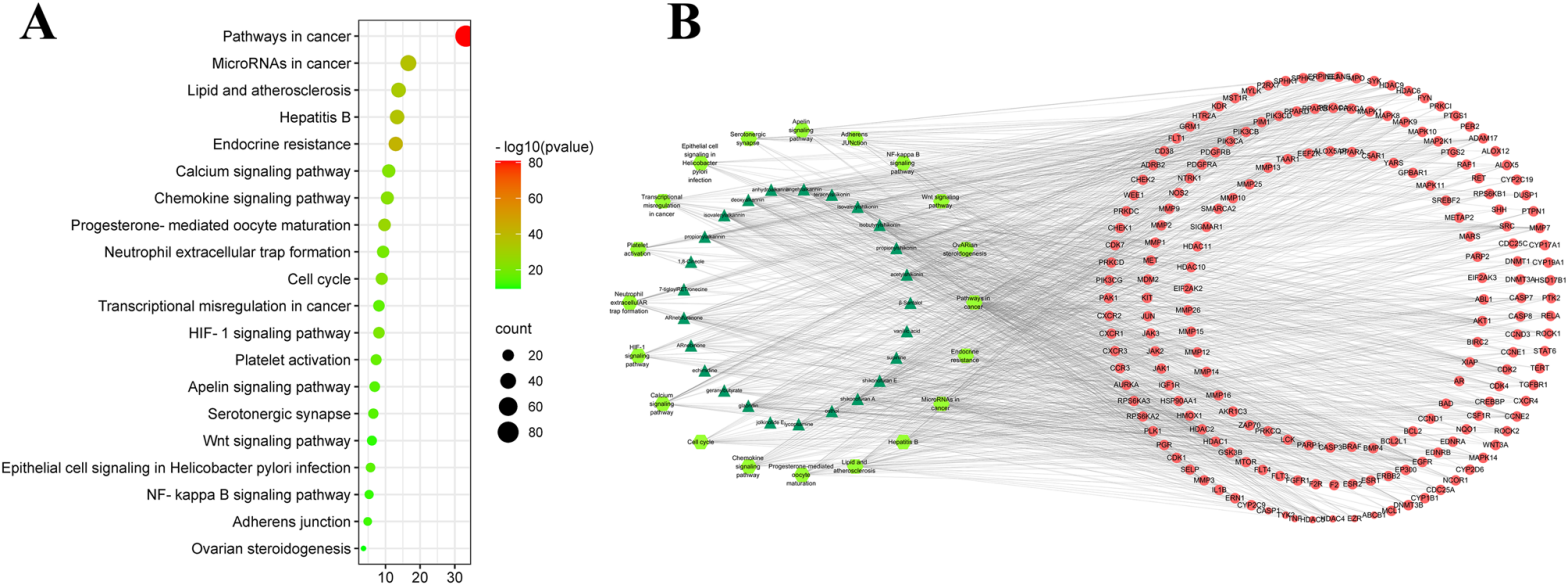
(**A**) Analysis of the anti-cancer KEGG pathway in *Lithospermum*; (**B**) Anticancer Chemical Composition—Target—Pathway Network of Lithospermum.

**Figure 7:**
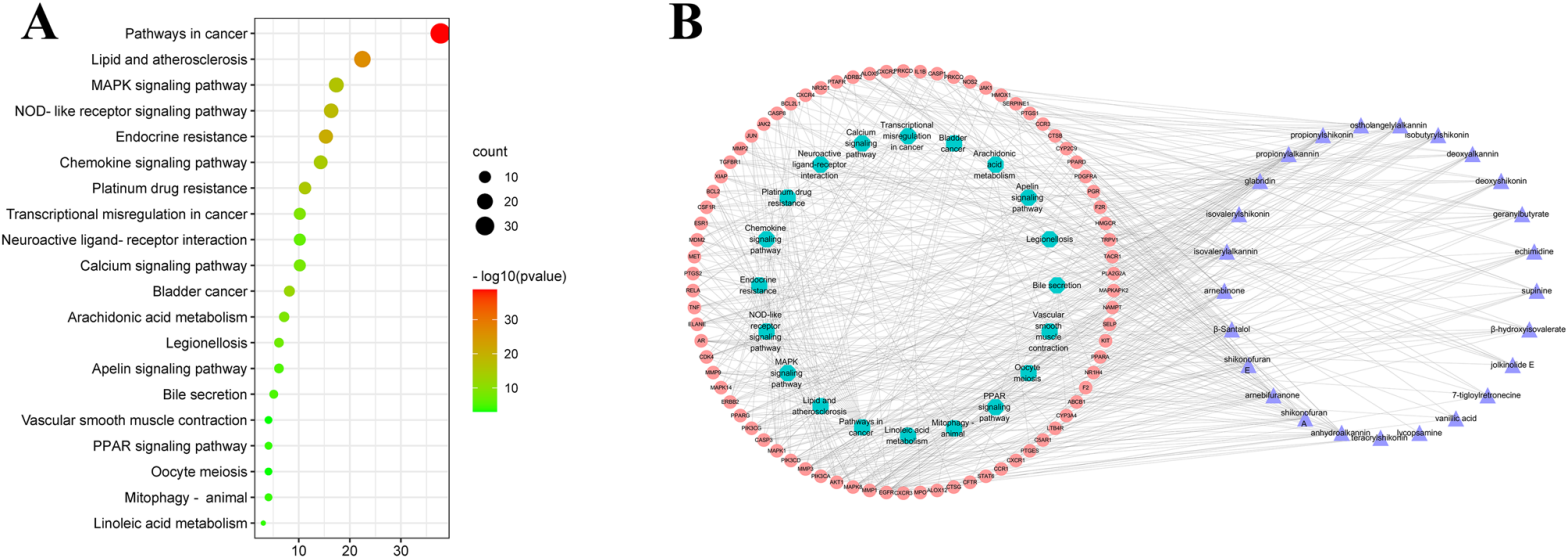
(**A**) Analysis of the anti-inflammatory KEGG pathway in *Arnebia*; (**B**) Anti-inflammatory Chemical Composition—Target—Pathway Network of *Arnebia.*

The Metascape database was used to analyze the Kyoto Encyclopedia of Genes and Genomes (KEGG) pathway of 247 core targets of the anticancer effects of *Lithospermum*, 98 core targets of the anti-inflammatory effects of *Arnebia*, and 156 core targets of the anti-anxiety effects of *Echium*. The first 20 most significant pathways upon visual analysis are shown in Figs. 5, 6, 7B. The findings revealed that the signaling pathways closely related to the anticancer effect of *Lithospermum* primarily included pathways involving microRNAs in cancer and so on. The signaling pathways closely related to the anti-inflammatory effect of *Arnebia* mainly include the NOD-like receptor and mitogen-activated protein kinase (MAPK) signaling pathways. The signaling pathways closely related to the anti-anxiety effect of *Echium* mainly include the serotonergic synapse and neuroactive ligand-receptor interaction pathways. The “active ingredient-target-pathway” network was further constructed. The results are shown in Figs. 5, 6, 7A.

## Discussion

Lithospermeae, originating from the Mediterranean and Western Asia, has a long history of medicinal use. Previous studies have found that the four most diverse genera of Lithospermeae changed during Miocene, with *Alkanna* and *Onosma* showing considerable diversity, *Lithospermum* further dispersing into North America, South America, and Africa, and *Echium* dispersing into Macaronesia^28^. Lithospermeae has the largest diversity in semi-arid to arid habitats and has an obvious overall diversity center in western Eurasia and the Mediterranean Basin^8^, consistent with our results. Lithospermeae is rich in red shikonin naphthoquinones, and the naphthoquinones in most genera are shikonin or alkannin. The main nucleus of shikonin is 5,8-dihydroxy1,4-naphtoquinone and has an isohexenyl side chain, and the difference in its configuration comes from the chiral carbon atom on the side chain^29^. These compounds have good biological activity and are the main substances in the pharmacological effects of Lithospermeae. It has been well-established that the chemical constituents of plants are closely related to their efficacy and pharmacological activity. Different groups of medicinal plants and different parts of the same medicinal plant may have different varying traditional efficacy.

The molecular phylogeny, geographical distribution, chemical composition, traditional application, and pharmacological effects of Lithospermeae were explored with respect to its pharmaphylogeny. Following the genetic theory, Lithospermeae plants are divided into three groups. Group one consists mainly of *Onosma*, followed by *Maharanga*, group two consists of *Lobostemon* and *Echium*, and group three consists of *Alkanna*, *Arnebia*, *Stenosolenium*, *Lithodora*, *Lithospermum*, *Buglossoides*, and *Glandora*.

Group 1: *Onosma* and *Maharanga*. While only a few studies on *Maharanga*, *Maharanga* has been used in traditional applications for constipation^17^, with antibacterial and antiviral pharmacological activities^30^. *Onosma* was mainly distributed on the Mediterranean coast and Tibet in China. The roots of *Onosma* were often reported to have been used as medicine for clearing heat and cooling blood, detoxification, treatment of rashes, and respiratory diseases^17^. The petals of *Onosma* were used for heart disease and rheumatic diseases. The main chemical constituents of *Onosma* are shown in Table 1, and its unique chemical components make its pharmacological activity remarkable. *Onosma* contains alkannins, shikonins, flavonoids, ferulic acids, and vanillic acids, which have been responsible for anti-inflammatory, wound healing, analgesic, and antibacterial activities (Tables 1, 2). Cypriots commonly used *Onosma fruticosa* for respiratory diseases^17^. The activity of the crude extract of *Onosma bracteatum* has shown promising results for asthma^31^.

Group 2: *Echium* and *Lobostemon*. *Echium* is mainly distributed along the Mediterranean coast and in western North America. *Echium* is often used as whole herb. It can be seen from Table 2 that in the traditional application, it is used to treat rheumatic pain, abscess, respiratory tract infection, trauma, exterior relief, and diuresis. Particularly, its petals are used for antianxiety, sedation, and cold relief^17^. Table 1 depicts that the *Echium* primarily contains shikonins, phenolic acids, and pyrrolizidine alkaloids. Chemical components of *Echium* were notably associated with its traditional applications and pharmacological effects. Shikonins are often used for their anti-inflammatory, antibacterial, antitumor, wound healing effects, and so on^32^. Phenolic acids have certain anticancer properties and are known to promote blood circulation^33^. The activity of the crude extract of *Echium amoenum* has been found to have anti-anxiety effects^34,35^, which was consistent with its traditional medicinal use. In previous studies, shikonin has been found to have a protective effect on the nervous system^36^, but the active ingredients responsible for its anti-anxiety effects are not clear. In this study, we used network pharmacology to analyze the molecular association between the chemical composition and anti-anxiety effects of *Echium* to explain its overall mechanism of action. In addition to shikonin, results of network pharmacology revealed the presence of caffeic acid, isobutyrylshikonin, and other compounds involved in the serotonergic pathway through targets such as monoamine oxidase B and APP. Compounds such as acetylshikonin and 2-methyl-n-butyrylshikonin regulate neuroactive ligand-receptor interaction pathways through targets such as recombinant adenosine a1 receptor (ADORA1) and adenosine a2a receptor (ADORA2A), allowing exertion of anti-anxiety effects (Fig. 5A,B).

*Lobostemon* is in the southwest corner of Australia and is mainly used for bacteriosis, ulcers, and wounds in traditional applications^17^. The chemical constituents of *Lobostemon* were mainly phenolic acids, flavonoids, and alkaloids. Clinically, it is mainly used for its anti-HIV and wound healing effects, etc.^24,37^. At present, there are only a limited number of reports on *Lobostemon,* and more studies are needed to better explore its pharmaphylogeny.

Group 3: *Alkanna*, *Arnebia*, *Stenosolenium*, *Lithodora*, *Lithospermum, Buglossoides*, and *Glandora*. Group 3 was further divided into three parts. *Alkanna* and *Lithodora* were first discussed, followed by *Buglossoides*, *Glandora*, and *Lithospermum*, and finally, *Arnebia* and *Stenosolenium*.

Part 1: *Alkanna* and *Lithodora. Lithodora* is mainly distributed on the Mediterranean coast and has been traditionally used for blood purification, chilblains, colds, cough, enteritis, fever, high blood pressure, and liver protection^17^. However, there are limited studies on its chemical constituents, with only aliphatic and ester compounds being reported (Table 1).

*Alkanna* is mainly distributed along the Mediterranean coast and the western coast of North America. In traditional applications, its roots are often used to treat diarrhea, abscess, wounds, burns, sore throat, etc.^17^. It mainly consists of quinone compounds, but phenolic acids, alkaloids, and flavonoids have also been found in *Alkanna*. Pharmacological studies have shown that in addition to analgesic, antibacterial, anticancer, antibacterial, anti-inflammatory, antioxidant, and wound healing effects, it also has benefits against nociceptive pain and cutaneous leishmaniasis (Table 2).

Part 2: *Buglossoides, Glandora*, and *Lithospermum. Lithospermum erythrorhizon* is one of the commonly used Chinese herbal medicines^38^. *Lithospermum* diversity notably changed during the Middle Ages, with further dispersals of *Lithospermum* into North America, South America, and Africa observed^8^. In this study, it was found that *Lithospermum* was geographically limited to Asia. The results are inconsistent with previous reports, which may be due to the small sample size and the need to increase the sample information for further analysis. In addition to the common functions of Lithospermeae of clearing heat and detoxification, traditional applications for coughing, anti-inflammatory, promoting blood circulation, analgesia, and tonification for weakness have also been reported. It has been used as a diuretic and contraceptive in Europe and India, and its fruit is also known to assist in digestion^17^. *Lithospermum* mainly contains shikonins, alkannins, and pyrrolizidine alkaloids (Table 1). *Lithospermum* plants have antibacterial, anti-inflammatory, and antithrombotic effects, as well as the ability to palliate skin diseases (Table 2). Concurrently, crude extracts of *Lithospermum erythrorhizon* have been reported to assist in weight loss^39^. *Lithospermum ruderale*, mainly native to India, has also exhibited anti-gonadotropic activity^40^. From Fig. 1, *Lithospermum canescens* and *Lithospermum ruderale* can be seen to have similar sequences, but *Lithospermum canescens* has not been reported to have a contraceptive effect^17,40^. This highlight how genetic background is not the only determinant of the pharmacologic effects of medicinal plants. The potential of environmental factors in changing plant constituent’s merits further study. In previous studies, shikonin and its derivatives were observed to inhibit the growth of tumor cells by activating miR-17-5p/phosphatase and tensin homolog deleted on chromosome ten (PTEN)/protein kinase B (Akt), signal transducer and activator of transcription 3 (STAT3), phosphatidylinositol 3 kinase (PI3K)/Akt, and reactive oxygen species (ROS), among other pathways^32^. Through network pharmacology, we identified the signaling pathways closely related to the anticancer effect of *Lithospermum*. These pathways had the most enrichment targets, far more than others, and were mainly regulated via anhydroalkannin, arnebifuranone, and other compounds by regulating matix metalloproteinases1 (MMP1), recombinant cyclin dependent kinase 2 (CDK2), and epidermal growth factor 2 (EGF2). These findings provide a direction for *Lithospermum* as an antitumor drug (Fig. 6A,B).

*Buglossoides* and *Glandora* are relatively less studied. *Buglossoides* is produced in Asia, Europe, and North Africa. It is traditionally used for weight loss and has the biological activity of antioxidants^17^. Aliphatic and ester compounds have been identified in chemical studies. *Glandora* is native to North Africa and South Europe. It is worth noting that the synonym of *Glandora diffusa* in *Glandora* is *Lithospermum diffusum* and *Lithodora diffusa*^41,42^. In traditional applications, *Glandora* is used for its diuretic, depurative, and antihypertensive effects. It contains phenolic acids, flavonoids, fatty acids, etc., known to have a certain anticancer effect (Tables 1, 2).

Part 2: *Arnebia* and *Stenosolenium. Arnebia* is mainly distributed in northern Africa and western Asia. *Arnebia guttata* and *Arnebia euchroma* are the source of authentic Chinese medicinal materials of ‘Zicao’^38^. In traditional applications, it is often used for heat clearance, detoxification, burns, heart disease, skin diseases, blood circulation promotion, etc.^17^. *Arnebia* contains numerous quinone compounds, mainly shikonins and alkannins. It is commonly used for its anti-inflammatory, antibacterial, antioxidant, anticancer, and antithrombotic effects (Tables 1, 2). In addition to shikonin, polysaccharides in certain species have also been observed to exert antitumor effects. The crude extract has been found to inhibit the growth of human breast cancer cell line (MCF-7) cells and reduce the level of estrogen and progesterone in mice to achieve its anti-reproductive effect^43^. This seems to be consistent with the contraceptive effect of *Lithospermum*. The *Arnebia* species is often used for a variety of inflammatory conditions, such as arthritis, colitis, dermatitis, and so on^32^. Through network pharmacology, *β*-santalol, osthol, and arnebifuranone in species of *Arnebia* were identified to regulate the NOD-like receptor signaling pathway through recombinant mitogen activated protein kinase 8 (MAPK8), X-linked inhibitor of apoptosis protein (XIAP), mitogen-activated protein kinase 14 (MAPK14), and tumor necrosis factor (TNF) targets. Isovalerylshikonin, isovalerylalkannin, and arnebifuranone et al. regulate the MAPK signaling pathway through epidermal growth factor receptor (EGFR), MAPK8, and recombinant human protein kinase (AKT1) targets, exerting anti-inflammatory effects. Previous studies on the anti-inflammatory effect of *Arnebia* have identified MAPKs, TNF, and AKT as targets and MAPK as the signaling pathway. In this study, *Arnebia* was also found to regulate NOD-like receptor signaling pathways through XIAP targets. Although requiring further verification, this provides a new idea for harnessing the anti-inflammatory effects of *Arnebia* (Fig. 7A,B).

*Stenosolenium* is mainly distributed in eastern Asia. In traditional applications, the whole herb was used for dispelling wind, removing dampness, and managing joint pain. Meanwhile, the root has the effects of clearing heat, cooling blood, stopping bleeding, and relieving cough^17^. *Stenosolenium* contains quinones, phenolic acids, and pyrrolizidine alkaloids (Table 1). Further research is needed to better explore the pharmaphylogeny of *Stenosolenium*.

*Alkanna*, *Lithospermum*, *Arnebia*, *Echium*, and *Onosma* are the main sources of medicinal plants of Lithospermeae. Following palynology research, *Echium* and *Onosma* have a close genetic relationship. The chemical constituents of *Echium* and *Onosma* have similar traditional efficacy and pharmacological effects, suggesting their close relationship, which is consistent with the conclusion based on the ITS sequence. *Lithospermum* and *Arnebia* also have many similar characteristics as the main sources of medicinal ‘Zicao’. For instance, they have similar efficacy in traditional applications (Table 2). In the current study, they were found to have a contraceptive effect. Their chemical constituents are mainly quinones, and their growth areas are similar, mainly in Asia. During the phylogenetic process, *Lithospermum* and *Arnebia* originate from the same evolutionary branch. Therefore, the consistency of plant efficacy between different groups suggests a subsequent relationship between the two in terms of chemical composition and phylogenetic development. This has important practical value for finding new drug sources by exploring the genetic relationship between plants.

At present, there are only a few studies on *Lobostemon.* However, following the surface characteristics of the stigma and the sinkage of the stem tip, *Lobostemon* has been reported to be similar to *Echium*^4^. *Lithodora* and *Glandora* are perennial shrubs with flowers on top of leafy branches. The flowers of the two genera are both linear and petal-shaped, forming tubes. They have five small stamens in the corolla tube. *Glandora* is a species that evolved from *Lithodora*^42,45^. Therefore, it is speculated that *Lithodora* and *Glandora* have a close genetic relationship. *Buglossoides arvense* originally belonged to the *Lithospermum* plant, and the ecological suitability and geographical locations of the two genera were also similar. Following the existing data, it is speculated that *Buglossoides* may have a close relationship with *Lithospermum*.

This study summarizes the chemical constituents, traditional applications, and pharmacological effects of Lithospermeae medicinal plants. Moreover, this paper reports on the genetic relationship among Lithospermeae through molecular phylogeny, species distribution model, and network pharmacology. At present, studies on Lithospermeae mainly focus on taxonomy and systematic position relationship, and preliminary studies on chemical constituents and pharmacological activities have demonstrated the rich diversity and activity potential of Lithospermeae. Our study explored the intrinsic correlation of the medicinal value of Lithospermeae. Based on previous pharmaphylogeny studies, we included a species distribution model and ecological adaptability evaluation to provide research basics for the future cultivation of Lithospermeae species. Meanwhile, network pharmacology was added to predict the targets and pathways of related diseases. Natural medicinal plants are gaining popularity among researchers due to their high efficacy, high quality, and low side effects. However, due to the growth environment and human factors, numerous medicinal plants have been damaged. Therefore, it is particularly urgent to find suitable substitutes and suitable cultivation techniques. This study not only enriches the pharmacy theory, helps to better understand, and tap the medicinal value of Lithospermeae, but also provides a reference for the development of other medicinal plants. Unfortunately, the medicinal value of 470 species of Lithospermeae plants has not attracted much attention and in-depth research for a long time, and only a few species have been highlighted for their chemical and pharmacological activities. We need a large number of studies on medicinal plants of the Lithospermeae to confirm our assumption. Moreover, the internal relationship among Lithospermeae medicinal plants remains unclear. These areas are worthy of further in-depth studies in the future.

## Methods

### Plant systematics of Lithospermeae

Plant morphological characteristics can be found using http://www.iplant.cn/frps, CNKI, Pubmed, and Google scholar. (The data collected from 1975 to 2022.).

Phylogenetic studies mainly reflect the evolutionary relationship between species by constructing phylogenetic trees among species, to provide a basis for exploring the evolution of related organisms. Molecular systematics is the construction of a phylogenetic tree using deoxyribonucleic acid (DNA). It is believed that the posterior probability greater than 90% indicates that branches are significantly supported. The ITS sequences of nucleotide were downloaded from National Center for Biotechnology Information (NCBI) (https://www.ncbi.nlm.nih.gov/) and analyzed by MEGA11 software. The phylogenetic tree was constructed by the Test Neighbor-Joining Tree method. No. of Bootstrap Replications was set to 1000, Substitutions Type was Nucleotide, Model/Method selected Kimura 2-parameter model, Site Coverage Cutoff (%) was 90.

### Geographical distribution

The geographical distribution points of Lithospermeae, 8 genera, 30 species, distribution points of 219,890 collected from the GBIF database are saved as. csv format in the order of longitude and latitude. Meanwhile, 19 environmental factors and 1 altitude factor worldwide were transformed into. asc format in ArcGIS 10.8 (https://desktop.arcgis.com/zh-cn/system-requirements/latest/arcgis-desktop-system-requirements.htm ). The distribution points of 219,890 for Lithospermeae species and 20 environmental variables (https://worldclim.org/ ) were imported into Maximum entropy (MaxEnt, https://biodiversityinformatics.amnh.org/open_source/maxent/ ). The MaxEnt model selects the Jackknife method to evaluate the contribution of variables and uses cross validate method to run 10 times. The output mode of the model is 'Logistic' to make the predicted results closer to the probability of Lithospermeae species distribution, and the other parameters are the default parameters. The predicted value of the model is between 0 and 1, and the ASCII format file is output. This file is imported into ArcGIS software, and the file in. tif format is output. The reclassify tool is used to classify the species distribution based on the default threshold of 0.5, and the potential distribution areas of each species of Lithospermeae in the world are obtained. Finally, the potential distribution grid data of each species are added to obtain the global potential distribution areas of each genus and whole family of Lithospermeae.

### Chemical constituents, traditional applications, and pharmacological activities

The chemical constituents, traditional applications, and pharmacological activities of Lithospermeae were summarized and sorted through CNKI, Pubmed, Google scholar, Wangfang Database, and the Library of congress. The collected Lithospermeae data were analyzed (The data collected from 1975 to 2022).

### Network pharmacology

#### Prediction of potential targets of drug components

We transformed each chemical component identified above into standard simplified molecular input line entry system (SMILES) through PubChem and screened the above chemical components with the SwissADME data platform. The screening conditions were DL (at least three yes in five items) and GI. Subsequently, these SMILES were imported into Swiss Target Prediction to predict targets of compounds. Species were selected as Homo sapiens with probability > 0 as the screening condition (Table 3).

#### Disease targets prediction

We selected the common genera of Lithospermeae and treated the disease. In GeneCards database (http://www.genecards.org/), OMIM (http://www.omim.org/) databases, disease targets were searched by keywords such as cancer, inflammation, and anxiety disorder, and targets with score ≥ 5 were selected as disease targets. Concurrently, the intersection targets of *Lithospermum* target and cancer target, *Arnebia* target and inflammation target, *Echium* target, and anxiety disorder target were screened via the Venny platform.

#### KEGG pathway analysis

Based on Metascape 3.5 database, Gene Ontology (GO) and KEGG enrichment analysis were performed on the intersection targets of *Lithospermum* and cancer, *Arnebia* and inflammation, *Echium* and anxiety disorder (Figs. 5, 6, 7A)^102,103^. The key biological functions and signaling pathways of *Lithospermum* anti-cancer, *Arnebia* anti-inflammatory, and *Echium* anti-anxiety were obtained with P < 0.05. Moreover, the data were visualized using the macrobiotic cloud platform. Finally, the “active ingredient—core target—pathway” association network was constructed (Figs. 5, 6, 7B).

## Supplementary Information

Supplementary Information.

## Data Availability

All data generated or analyzed during this study are included in this article and its supplementary information files.
