# Supplementary material for: Exploring pharmaphylogeny from multiple perspectives: a case study on Lithospermeae
Source: Sci Rep. 2023 May 11;13:7636. doi: 10.1038/s41598-023-34830-4 (PMC10175555; doi:10.1038/s41598-023-34830-4)
Supplement: Supplementary file 1 — Supplementary Information. [file 41598_2023_34830_MOESM1_ESM.docx]

Supplementary Material

**Supplementary Figures and Tables**

**Supplementary Figures**

**Map source** [**https://bbs.pinggu.org/thread-10882790-1-1.html**](https://bbs.pinggu.org/thread-10882790-1-1.html)


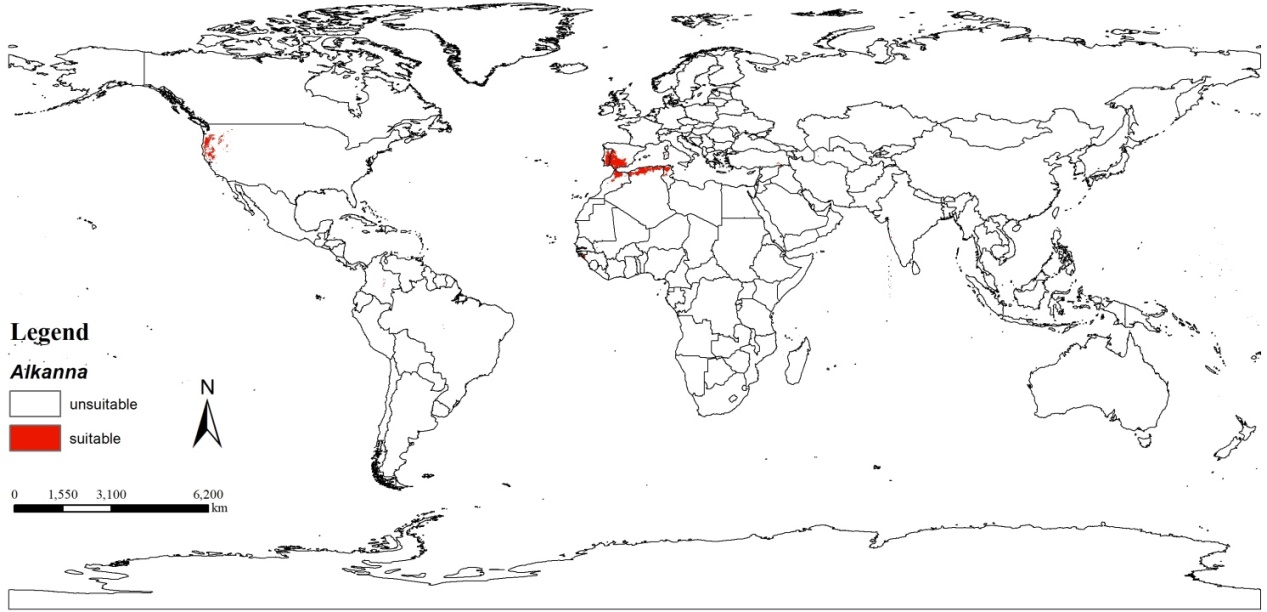


**Figures S1** Mainly distribution map of medicinal plants of *Alkanna***
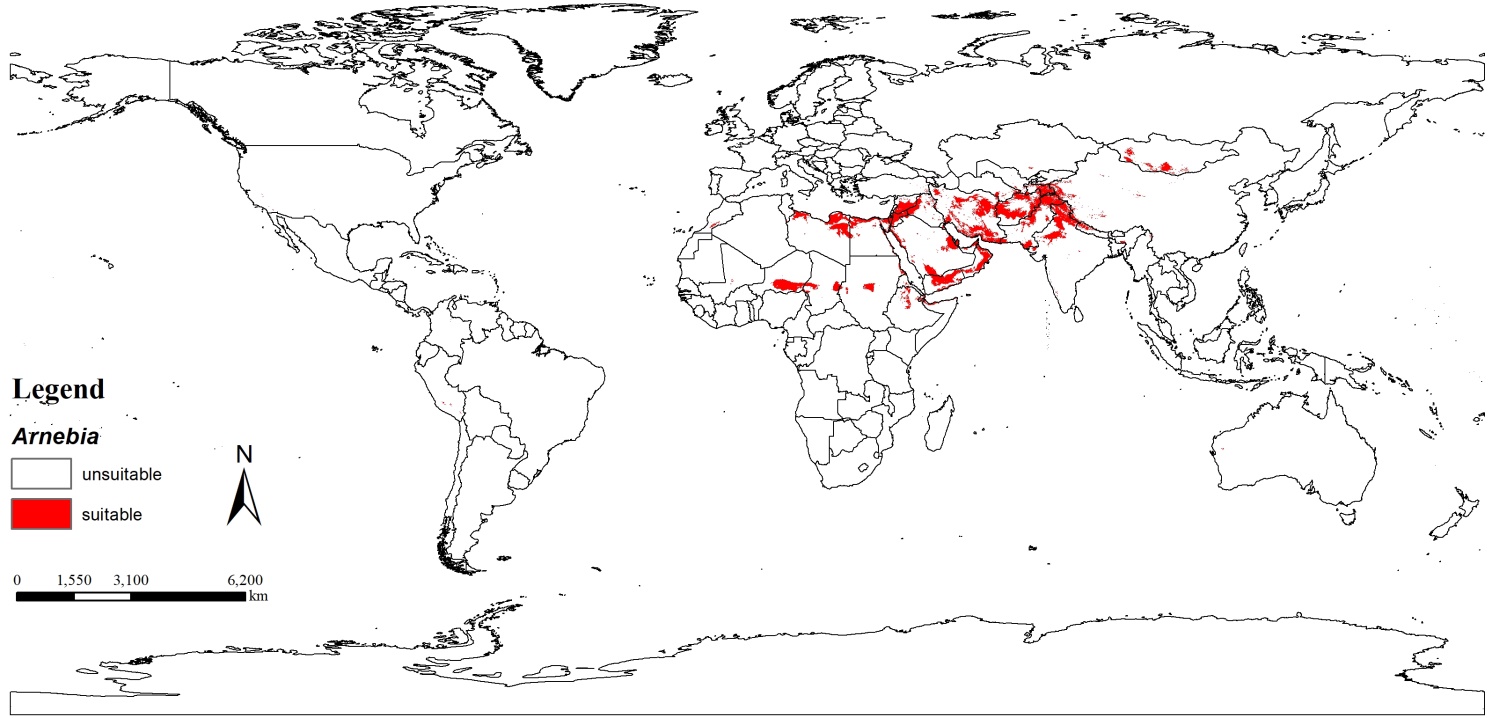
Figures S2** Mainly distribution map of medicinal plants of *Arnebia*


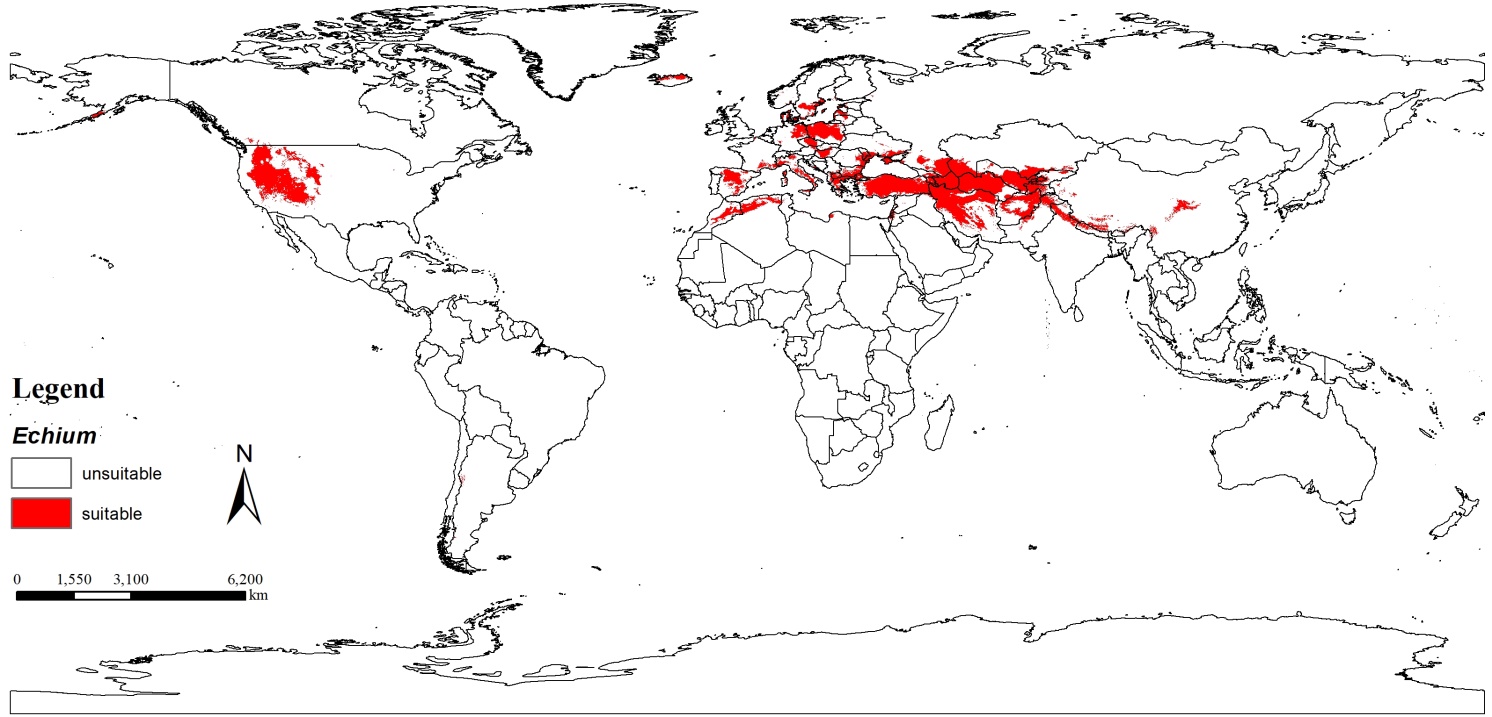
**Figures S3** Mainly distribution map of medicinal plants of *Echium*


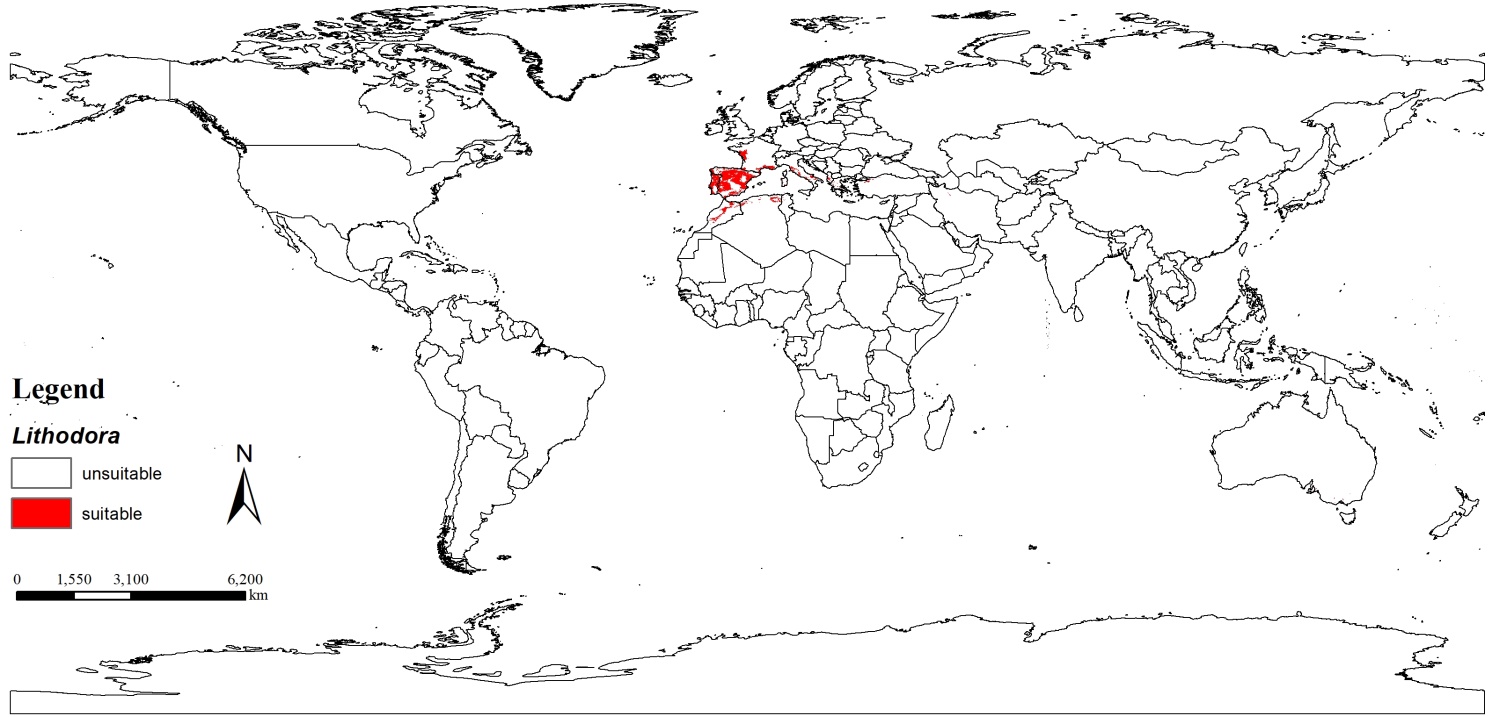
**Figures S4** Mainly distribution map of medicinal plants of *Lithodora*


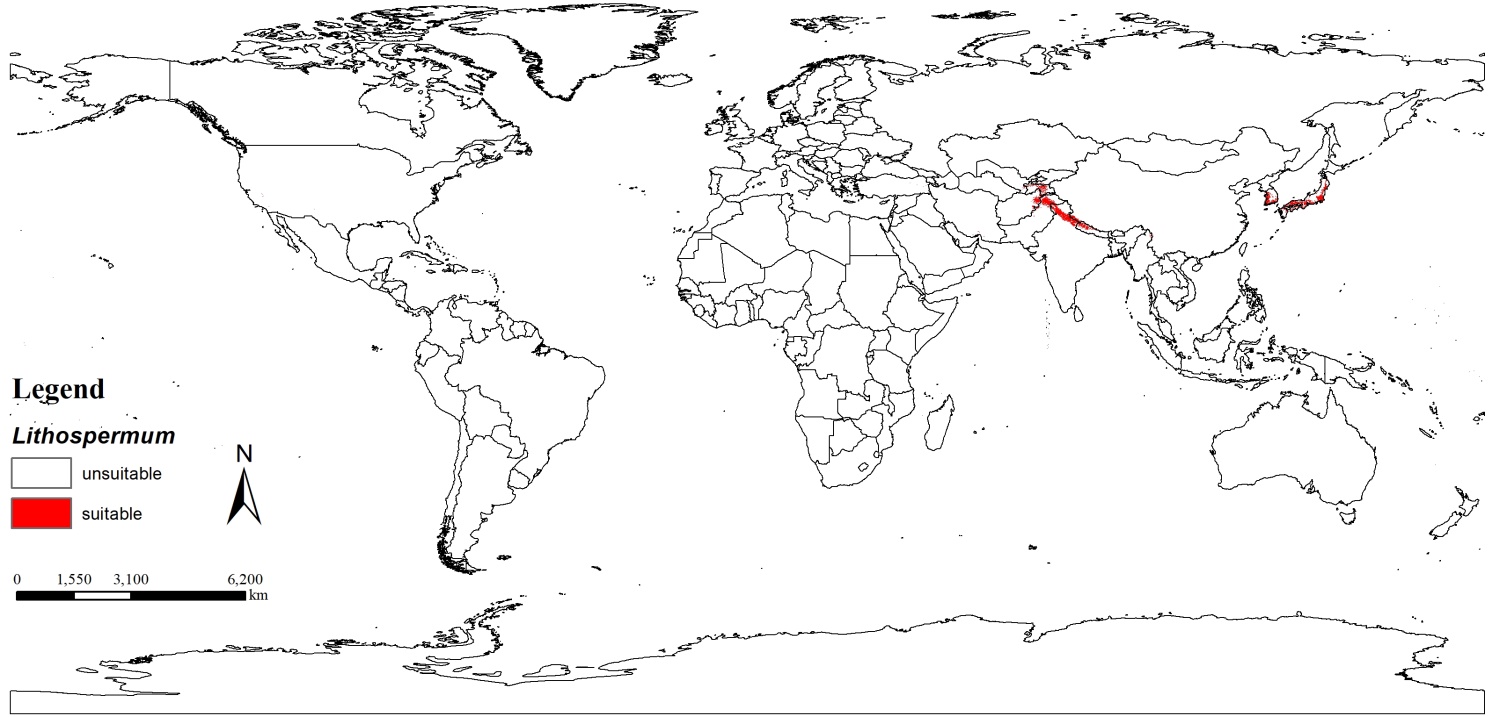
**Figures S5** Mainly distribution map of medicinal plants of *Lithospermum*


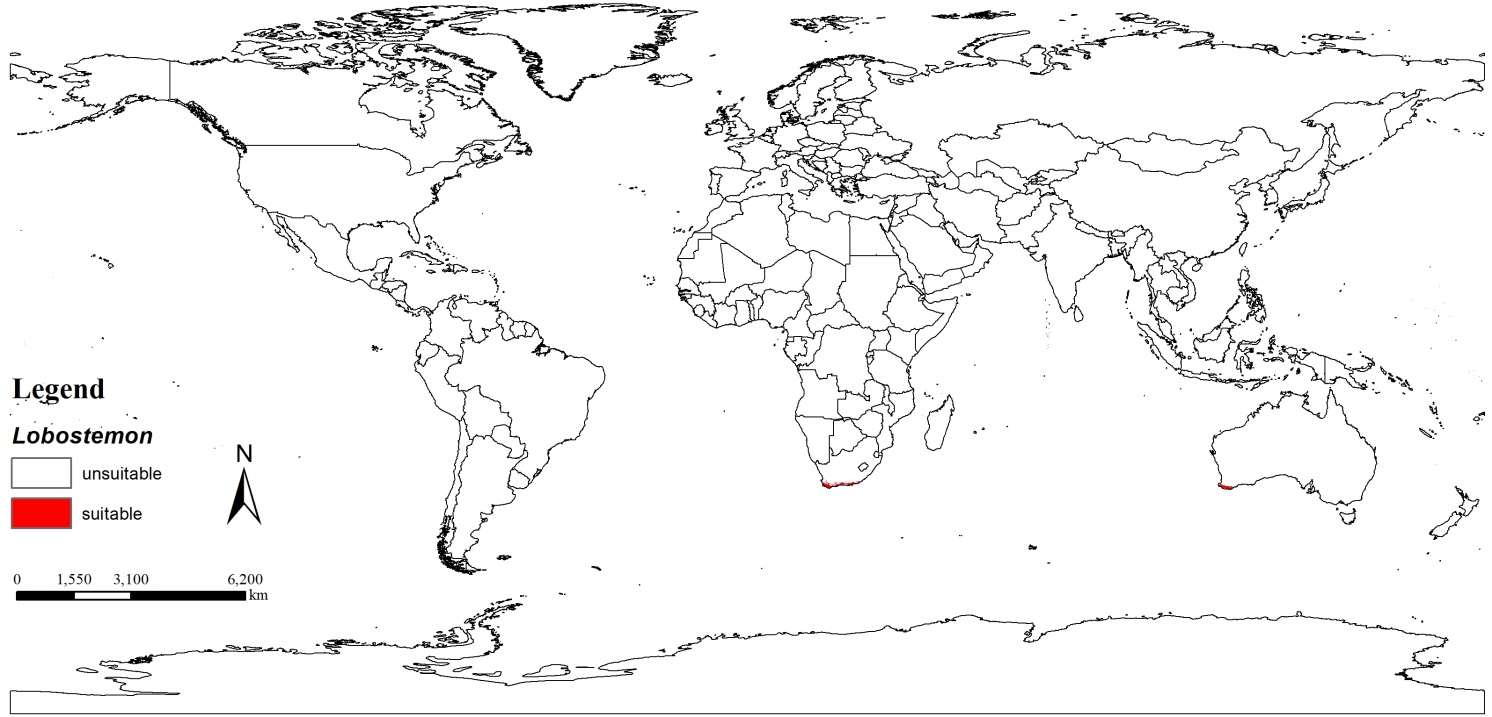
**Figures S6** Mainly distribution map of medicinal plants of *Lobostemon*


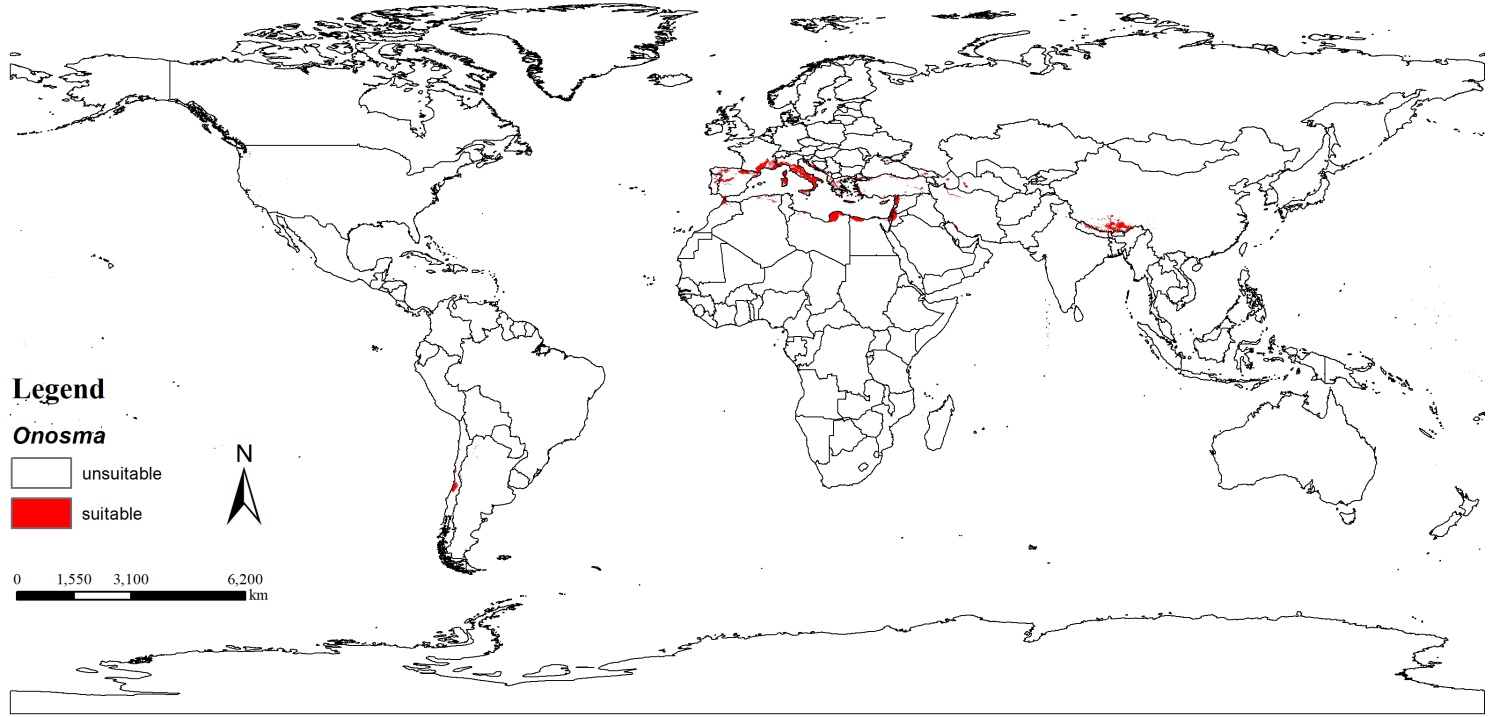
**Figures S7** Mainly distribution map of medicinal plants of *Onosma*


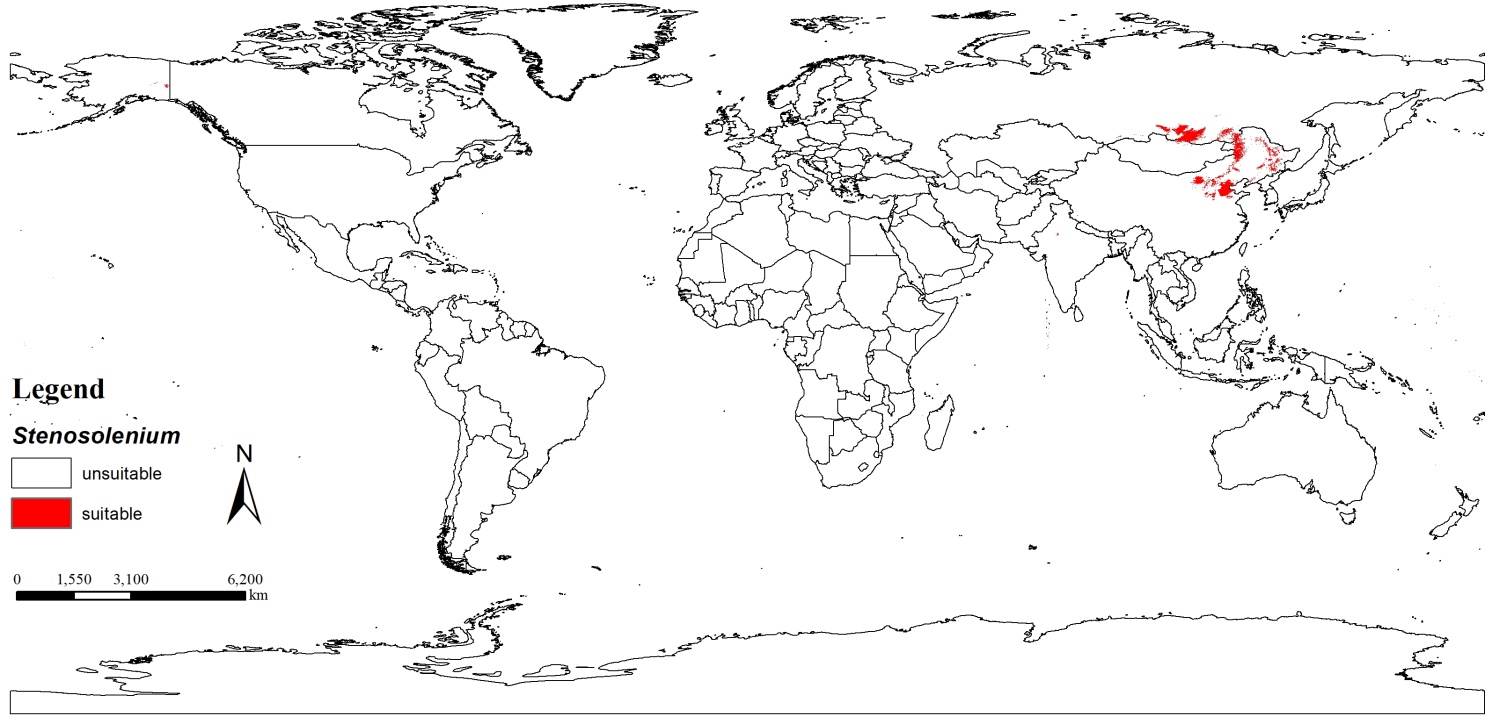
**Figures S8** Mainly distribution map of medicinal plants of *Stenosolenium*


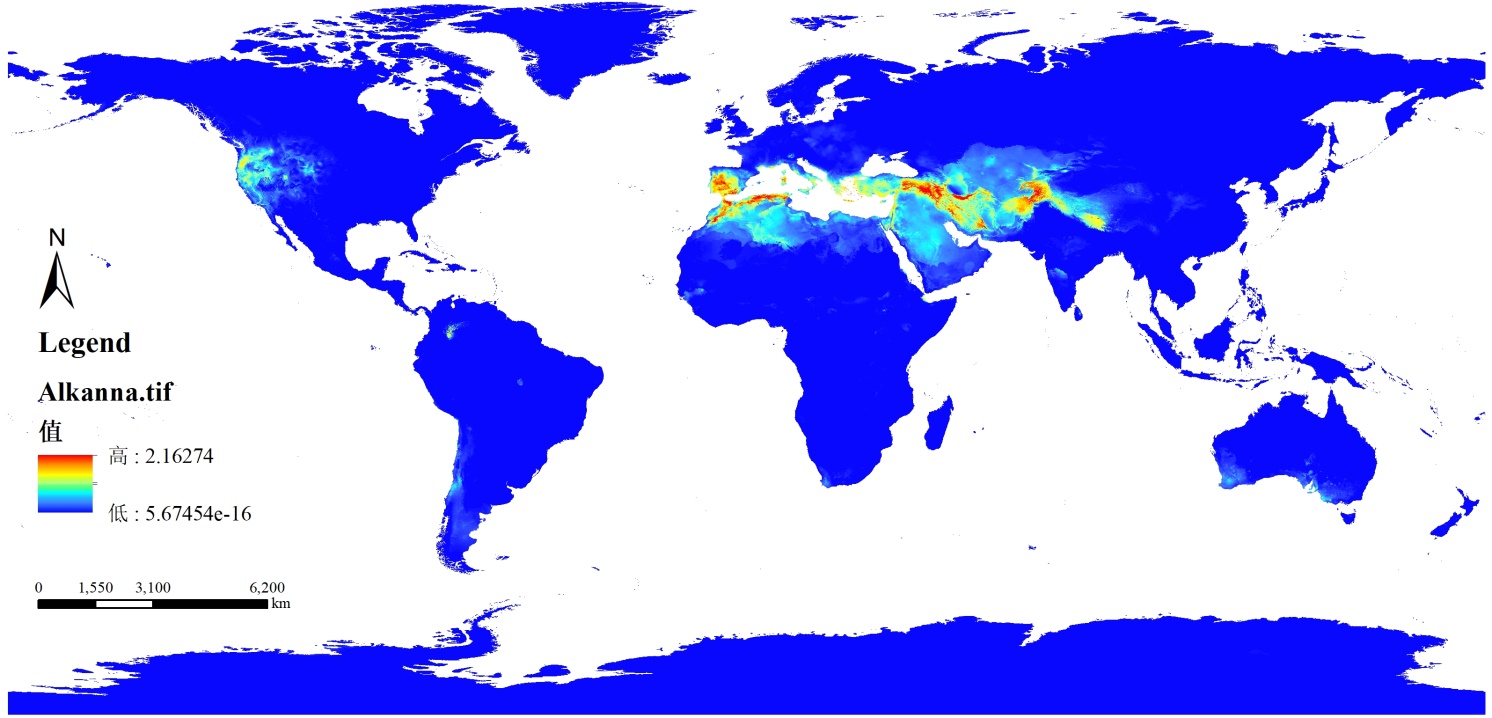
**Figures S9** Species adaptability distribution map of *Alkanna*


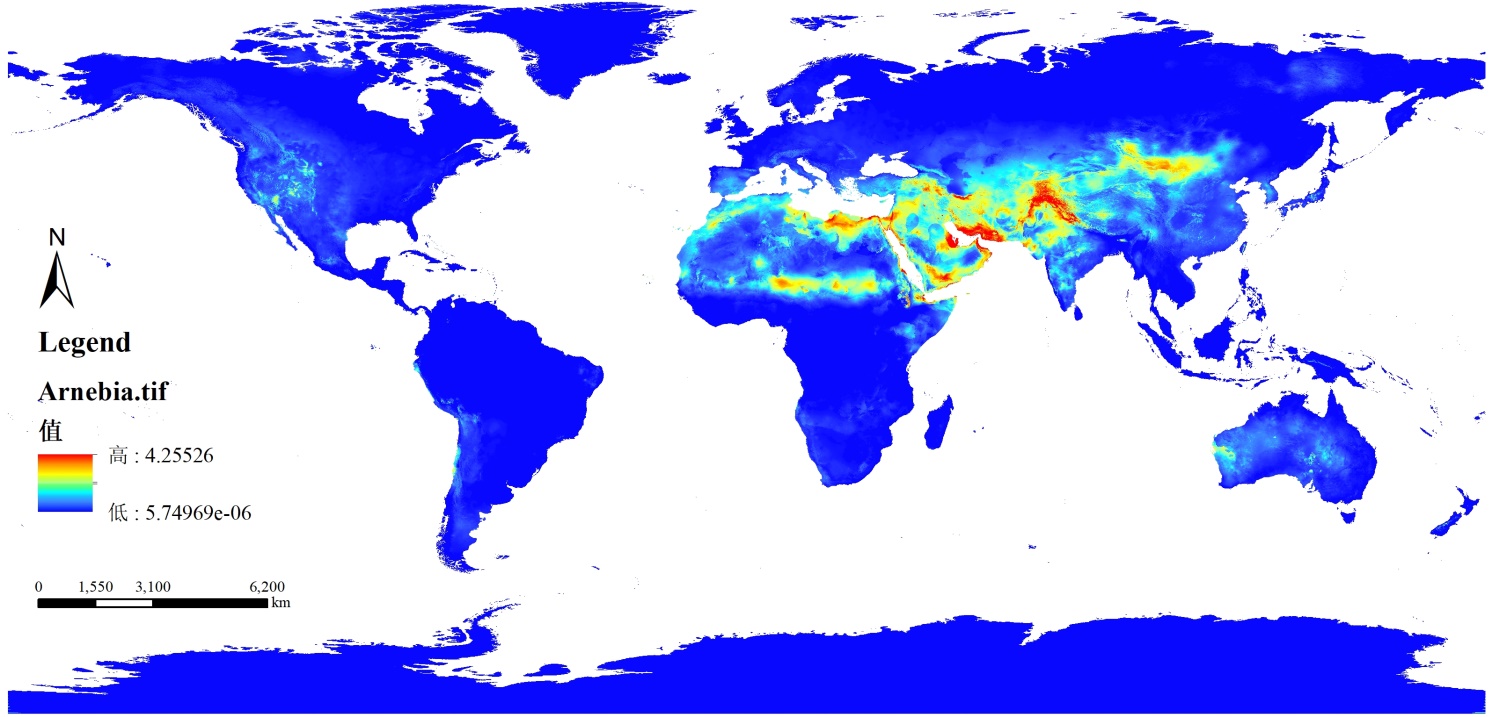
**Figures S10** Species adaptability distribution map of *Arnebia*


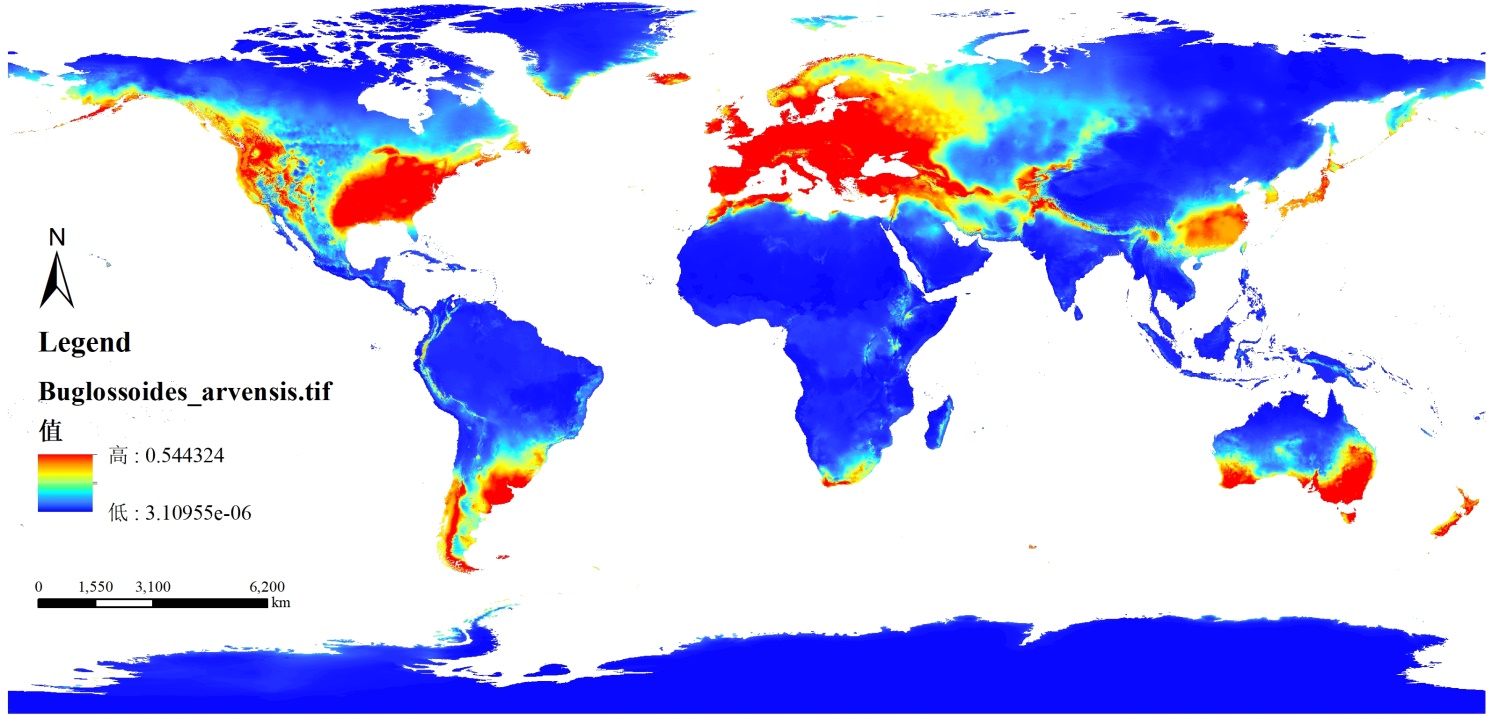
**Figures S11** Species adaptability distribution map of *Buglossoides arvensis*


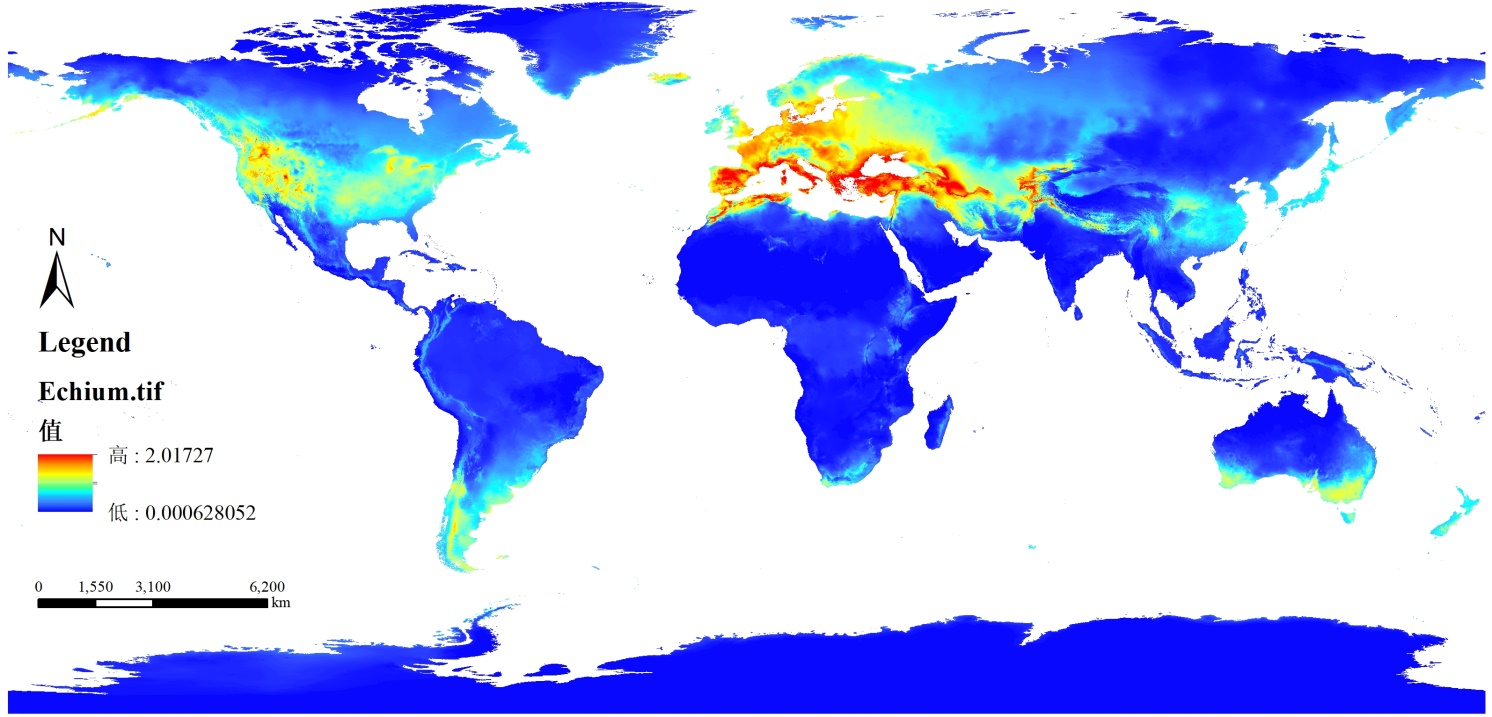
**Figures S12** Species adaptability distribution map of *Echium*


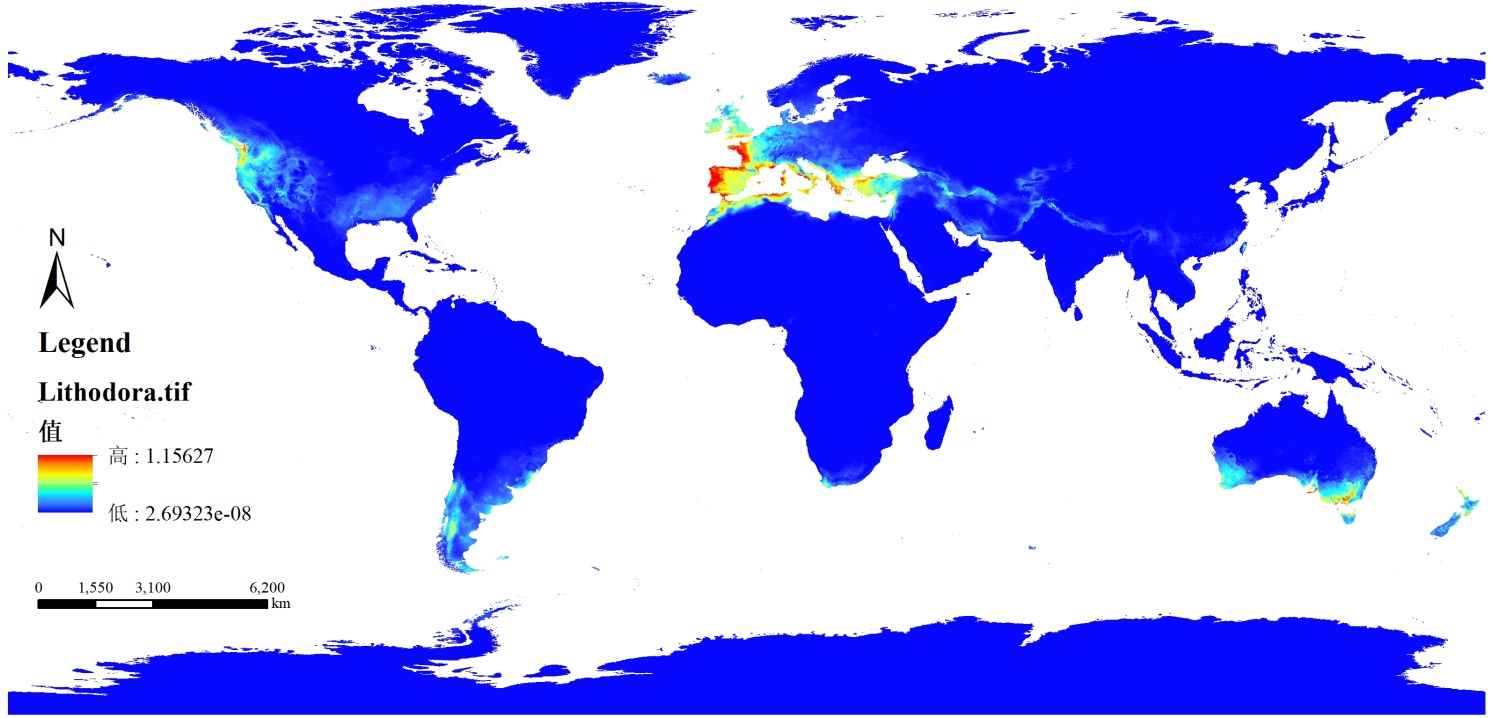
**Figures S13** Species adaptability distribution map of *Lithodora*


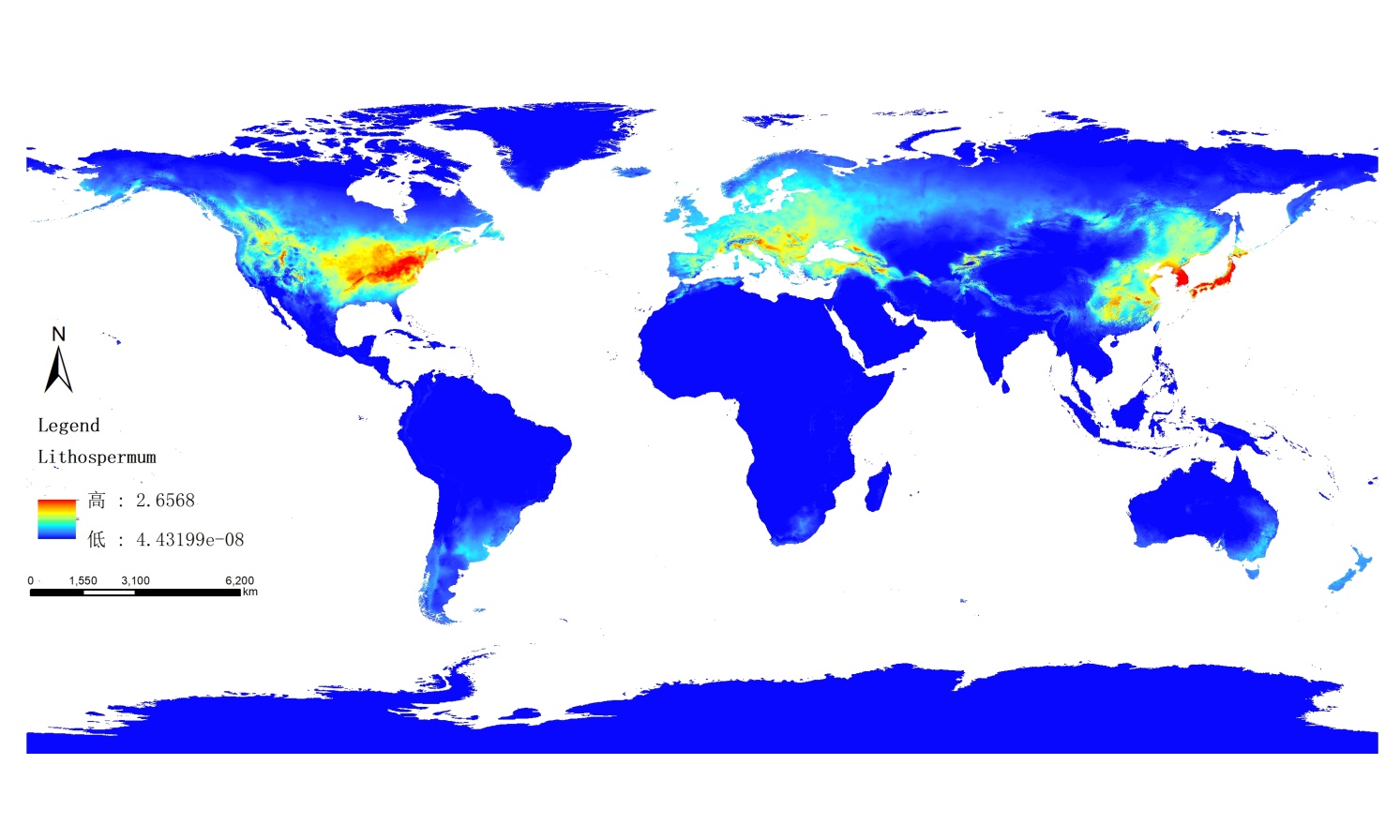


**Figures S14** Species adaptability distribution map of *Lithospermum*


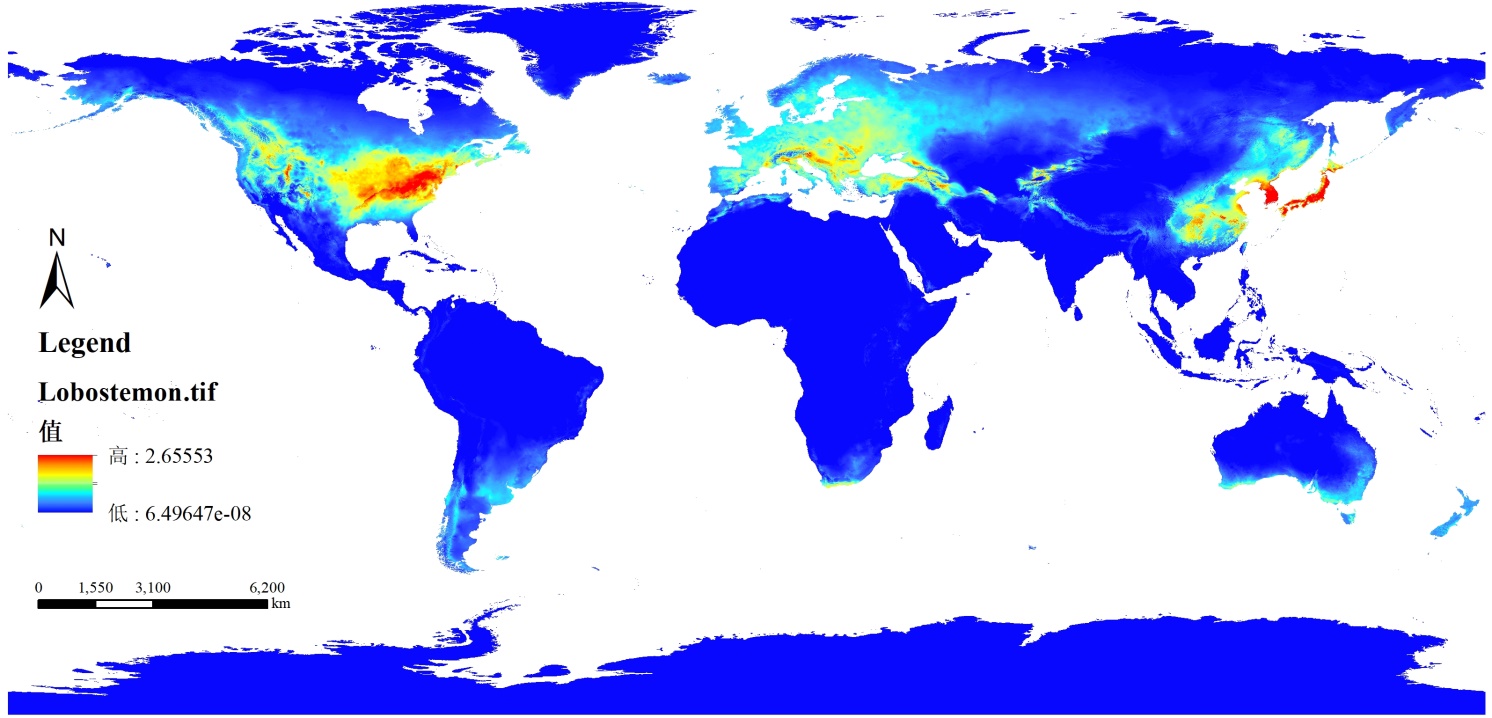


**Figures S15** Species adaptability distribution map of *Lobostemon*


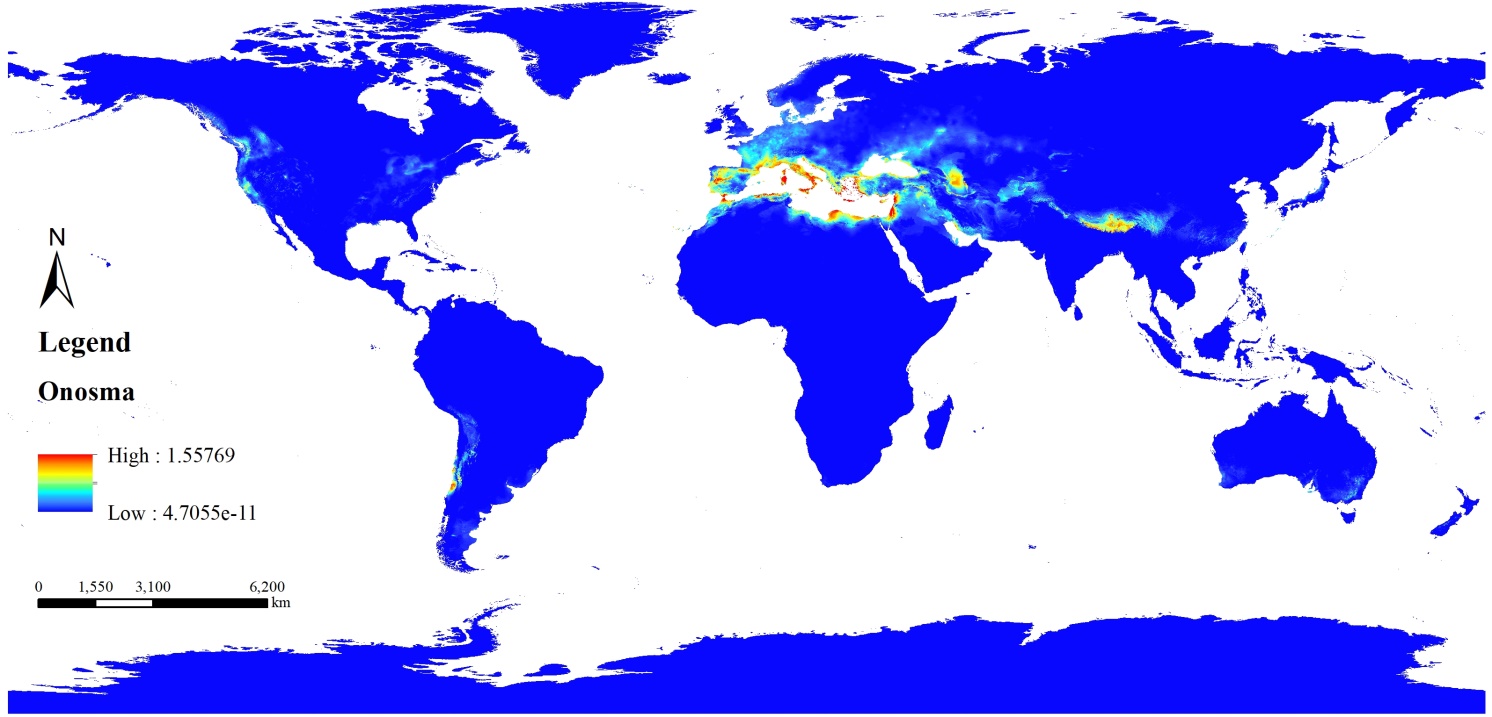


**Figures S16** Species adaptability distribution map of *Onosma*


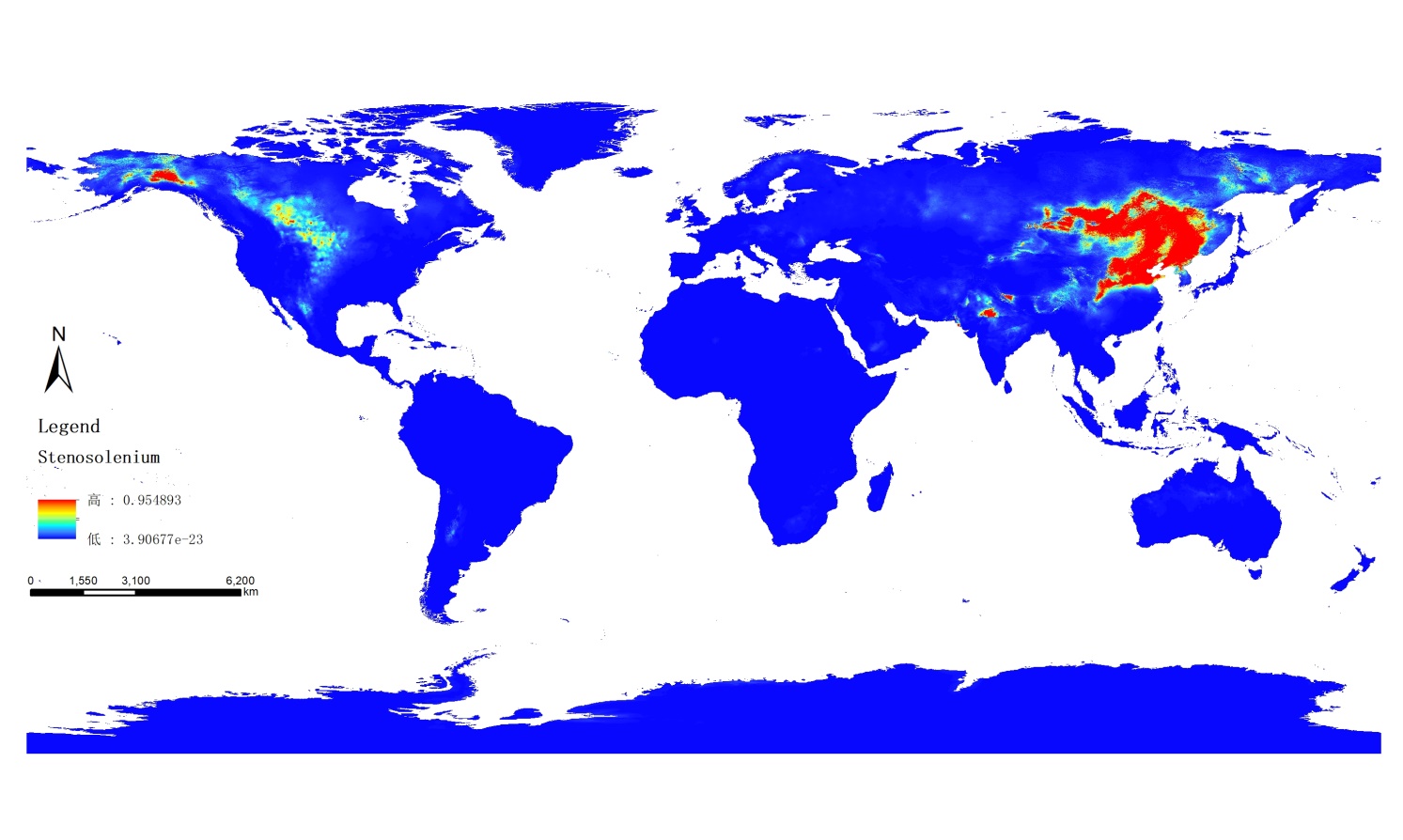


**Figures S17** Species adaptability distribution map of *Stenosolenium*

**Supplementary Table**

Table S1. Types of compounds contained in Lithospermeae

| Type of compounds | | Structures | Compounds | Genus | References |
| --- | --- | --- | --- | --- | --- |
| Quinones | Shikonins |  | shikonin | *Arnebia* , *Alkanna,Echium* , *Lithospermum* , *Onosma* , *Stenosolenium* | ^1-25^ |
|  |  |  | (E)4-hydroxy-2,4-dimethylpent-penteneacyl shikonin |  |  |
|  |  |  | (Z)2-Methylene-3-butenoyl shikonin |  |  |
|  |  |  | 1-methoxyacetyl shikonin |  |  |
|  |  |  | 2'-methyl-hexa-1',3'-dienoyl shikonin |  |  |
|  |  |  | 2-methyl-n-butyryl shikonin |  |  |
|  |  |  | 5-acetoxy-valeryl shikonin |  |  |
|  |  |  | acetylshikonin |  |  |
|  |  |  | lithoapermidin A |  |  |
|  |  |  | lithoapermidin B |  |  |
|  |  |  | butyrylshikonin |  |  |
|  |  |  | Dehydration shikonin |  |  |
|  |  |  | angelylshikonin |  |  |
|  |  |  | arnebin-6 |  |  |
|  |  |  | deoxyshikonin |  |  |
|  |  |  | 13-hydroxyisovaleryl shikonin |  |  |
|  |  |  | isobutyrylshikonin |  |  |
|  |  |  | isovalerylshikonin |  |  |
|  |  |  | methylshikonin |  |  |
|  |  |  | propionylshikonin |  |  |
|  |  |  | teracrylshikonin |  |  |
|  |  |  | benzoylshikonin |  |  |
|  |  |  | tigloylshikonin |  |  |
|  |  |  | valerylshikonin |  |  |
|  |  |  | α,α-dimethylpropionyl shikonin |  |  |
|  |  |  | α-methylbutyryl shikonin |  |  |
|  |  |  | α-methyl-n-butylshikonin |  |  |
|  |  |  | α-methylene-butenoyl shikonin |  |  |
|  |  |  | β,β-dimethylacryl shikonin |  |  |
|  |  |  | β-acetoxyisovaleryl shikonin |  |  |
|  |  |  | β-hydroxyisovaleryl shikonin |  |  |
|  |  |  | 2,3-dimethylpentenoyl-shikonin |  |  |
|  |  |  | cinnamoylshikonin |  |  |
|  |  |  | 3,4-methylenedioxy-cinnamoylshikonin |  |  |
|  |  |  | shikalkin |  |  |
|  | shikonin dimer |  | 6-(11′-deoxyalkannin)-alkannin/shikonin β-hydroxyisovalerylate | *Arnebia*, *Alkanna*, *Lithospermum* , *Onosma* | ^26-29^ |
|  |  |  | 6-(11′-deoxyalkannin)-alkannin/shikonin acetate |  |  |
|  |  |  | 6-(11′-deoxyalkannin)-alkannin/shikonin isobutyrylate |  |  |
|  |  |  | 6-(11′-deoxyalkannin)-alkannin/shikonin β,β-dimethylacrylate |  |  |
|  |  |  | dimer alkannin/shikonin |  |  |
|  |  |  | shikometabolins A |  |  |
|  |  |  | shikometabolins E |  |  |
|  |  |  | shikometabolin H |  |  |
|  |  |  | shikometabolins F |  |  |
|  |  |  | AE02-15-7 |  |  |
|  |  |  | AE02-15-8 |  |  |
|  |  |  | AE02-15-1 |  |  |
|  |  |  | AE02-15-2 |  |  |
|  | Alkannans |  | 2''-(S)-α-methylbutyryl alkannin | *Alkanna*, *Arnebia*, *Echium*, *Lithospermum*, *Onosma*, *Stenosolenium*,  *Maharanga* | ^7, 11, 20, 24, 30-42^ |
|  |  |  | β-hydroxyisovaleryl alkannin |  |  |
|  |  |  | β-hydroxyisovalerate alkannin |  |  |
|  |  |  | β-methoxylacetyl-alkanin |  |  |
|  |  |  | β-acetoxyisovalerylalkannin |  |  |
|  |  |  | β,β-dimethylacrylhydroxy alkannin |  |  |
|  |  |  | β，β-dimethylacrylate alkannin |  |  |
|  |  |  | β,β-dimethylacryl alkannin |  |  |
|  |  |  | α-methyl-n-butyl alkannin |  |  |
|  |  |  | teracrylalkannin |  |  |
|  |  |  | propionylalkannin |  |  |
|  |  |  | isovalerylalkannin |  |  |
|  |  |  | isobutyrylalkannin |  |  |
|  |  |  | dimethylacryl alkannin |  |  |
|  |  |  | deoxyalkannin |  |  |
|  |  |  | anhydroalkannin |  |  |
|  |  |  | angelylalkannin |  |  |
|  |  |  | alkannin |  |  |
|  |  |  | alkannan |  |  |
|  |  |  | arnebin-2 |  |  |
|  |  |  | acetylarnebin-2 |  |  |
|  |  |  | acetylalkannin |  |  |
|  |  |  | 5-methoxyangenyl alkannin |  |  |
|  |  |  | 5,8-O-dimethyl-11-deoxy alkannin |  |  |
|  |  |  | 3,4-(Methylenedioxy)cinnamoyl alkannin |  |  |
|  | Furans |  | shikonofuran A | *Arnebia*, *Lithospermum*, *Onosma* | ^43-46^ |
|  |  |  | shikonofuran B |  |  |
|  |  |  | shikonofuran C |  |  |
|  |  |  | shikonofuran D |  |  |
|  |  |  | shikonofuran E |  |  |
|  |  |  | hydroxyechinofuran B |  |  |
|  |  |  | echinofuran B |  |  |
|  |  |  | arnebifuranon |  |  |
|  |  |  | hydroxyshikonofuran A |  |  |
|  |  |  | hydroxyshikonofuran B |  |  |
|  |  |  | hydroxyshikonofuran C |  |  |
|  |  |  | hydroxyshikonofuran D |  |  |
|  |  |  | hydroxyshikonofuran E |  |  |
|  |  |  | hydroxyshikonofuran G |  |  |
|  |  |  | hydroxyshikonofuran H |  |  |
|  |  |  | hydroxyshikonofuran I |  |  |
|  |  |  | hydroxyshikonofuran J |  |  |
|  | Others |  | arnebinol | *Alkanna*, *Arnebia*, *Lithospermum*, *Onosma* | ^36, 47-50^ |
|  |  |  | shikonine |  |  |
|  |  |  | arnebinone |  |  |
|  |  |  | arnebifuranone |  |  |
|  |  |  | arnebinol B |  |  |
|  |  |  | des-O-methyllasiodiplod |  |  |
|  |  |  | thizonone |  |  |
|  |  |  | clavilactone A. |  |  |
|  |  |  | amebinol A |  |  |
|  |  |  | amebinol C |  |  |
|  |  |  | amebinol B |  |  |
|  |  |  | amebinol D |  |  |
|  |  |  | amebinone B |  |  |
|  |  |  | 9,17-epoxyamebinol |  |  |
|  |  |  | 2-methyl-5 [2',8' dihydroxy-1',4'naphth oquinonyl]-5-hydroxypenten-2-oic acid |  |  |
|  |  |  | alkanfuranol |  |  |
|  |  |  | anthraquinone Ⅰ |  |  |
|  |  |  | lithoapermidin C |  |  |
|  |  |  | lithoapermidin D |  |  |
|  |  |  | lithoapermidin E |  |  |
|  |  |  | lithoapermidin F |  |  |
|  |  |  | arnebidin |  |  |
|  |  |  | arnebacene |  |  |
|  |  |  | arnebiabinone |  |  |
|  |  |  | arnebin-5 |  |  |
|  |  |  | euchroquinols A |  |  |
|  |  |  | euchroquinols B |  |  |
|  |  |  | euchroquinols C |  |  |
| Alkaloids | Pyrrolizidine alkaloids |  | lithosenine | *Alkanna*, *Arnebia*, *Echium*, *Lithospermum*, *Onosma*, *Stenosolenium*, *Lobostemon* | ^5, 21, 30, 51-72^ |
|  |  |  | acetyllithosenine |  |  |
|  |  |  | hydroxymyoscorpine |  |  |
|  |  |  | myoscorpine |  |  |
|  |  |  | O7-angeloylretronecine |  |  |
|  |  |  | intermedine |  |  |
|  |  |  | O9-angeloylretronecine |  |  |
|  |  |  | europine |  |  |
|  |  |  | heliotrine |  |  |
|  |  |  | lycopsamine |  |  |
|  |  |  | echimidine |  |  |
|  |  |  | 3-O-Acetylheliosupine |  |  |
|  |  |  | 3'-O-acetylechimidine |  |  |
|  |  |  | 5'-O-acetylechimidine |  |  |
|  |  |  | 7- angeloylretronecine |  |  |
|  |  |  | 7-O-Acetylechinatine |  |  |
|  |  |  | 7-O-Acetyllycopsamine N-oxide |  |  |
|  |  |  | 7-O-acetylvulgarine |  |  |
|  |  |  | 9-angeloylretronecine |  |  |
|  |  |  | 9-O-angelylretronecine |  |  |
|  |  |  | 9-tigloylretronecine |  |  |
|  |  |  | acetylechimidine |  |  |
|  |  |  | canescine |  |  |
|  |  |  | echihumiline |  |  |
|  |  |  | echihumiline N-oxide |  |  |
|  |  |  | echimidine-N-oxide |  |  |
|  |  |  | echimiplatine |  |  |
|  |  |  | echiuplatine |  |  |
|  |  |  | heliosupine |  |  |
|  |  |  | jacozine |  |  |
|  |  |  | leptanthine |  |  |
|  |  |  | lycopsamine-N-oxid |  |  |
|  |  |  | monocrotaline |  |  |
|  |  |  | supinine |  |  |
|  |  |  | uplandicine |  |  |
| Phenolic acids | Caffeic acid tetramers |  | salvianolic acid E | *Arnebia*, *Glandora* | ^73-75^ |
|  |  |  | salvianolic acid B |  |  |
|  |  |  | rabdosiin |  |  |
|  | Caffeic acid trimer |  | lithospermic acid | *Lithospermum*, *Stenosolenium*, *Lobostemon*, *Glandora* | ^5, 76-79^ |
|  |  |  | lithospermic acid B |  |  |
|  |  |  | 9''-methyl lithospermate |  |  |
|  |  |  | 9'-methyl lithospermate |  |  |
|  |  |  | salvianolic acid A |  |  |
|  |  |  | salvianolic acid H |  |  |
|  |  |  | salvianolic acid I |  |  |
|  |  |  | salvianolic acid C |  |  |
|  | Caffeic acid dimer |  | rosmarinic acid | *Alkanna*, *Arnebia*, *Echium*, *Lithospermum*, *Onosma*, *Stenosolenium*, *Lobostemon*, *Glandora* | ^73, 80-86^ |
|  |  |  | chlorogenic acid |  |  |
|  | Others |  | oleanolic acid | *Arnebia*, *Echium*, *Lithospermum*, *Onosma*, *Stenosolenium*, *Lobostemon*, *Glandora* | ^5, 77, 80, 87-96^ |
|  |  |  | ferulic acid |  |  |
|  |  |  | vanillic acid |  |  |
|  |  |  | syringic acid |  |  |
|  |  |  | sinapic acid |  |  |
|  |  |  | quinic acid |  |  |
|  |  |  | p-hydroxybenzoic acid |  |  |
|  |  |  | p-coumaric acid |  |  |
|  |  |  | hydrocaffeic acid |  |  |
|  |  |  | caffeic acid |  |  |
|  |  |  | benzoic acid |  |  |
|  |  |  | 4-hydroxy-3-methoxy benzoic acid |  |  |
|  |  |  | 4-hydroxy-3-methoxy cinnamic acid |  |  |
|  |  |  | 3-,4-dihydroxyphenyllactic acid |  |  |
|  |  |  | 4-hydroxybenzoic acid |  |  |
| Aliphatic and ester compounds |  |  | α-linolenic acid | *Arnebia*, *Echium*,  *Onosma*, *Lithodora*, *Buglossoides*, *Lobostemon*, *Glandora*, | ^73, 97-101^ |
|  |  |  | γ-linolenic acid |  |  |
|  |  |  | 3 - acetyl oleanolic acid |  |  |
|  |  |  | arnebia acid |  |  |
|  |  |  | tetracosanoic acid |  |  |
|  |  |  | stearidonic acid |  |  |
|  |  |  | docosahexaenoic acid |  |  |
|  |  |  | eicosapentaenoic acid |  |  |
|  |  |  | linolenic acid |  |  |
|  |  |  | 12-Oxophytodienoate |  |  |
|  |  |  | β-Carotene |  |  |
|  |  |  | germacrene-D |  |  |
|  |  |  | tetracosyl ester |  |  |
|  |  |  | lauric |  |  |
|  |  |  | myristic |  |  |
|  |  |  | palmitic |  |  |
|  |  |  | linoleic |  |  |
|  |  |  | a-Linolenic |  |  |
|  |  |  | oleic |  |  |
|  |  |  | stearic |  |  |
|  |  |  | docosanoic |  |  |
|  |  |  | Octyl ferulate |  |  |
|  |  |  | Tetradecyl ferulate |  |  |
|  |  |  | Ethyl 9- (2', 5'- dihydroxyphenyl) nonanoate |  |  |
|  |  |  | Octadecanol |  |  |
|  |  |  | lignoceric acid |  |  |
| Flavonoids |  |  | 5,4'- dihydroxy-7-methoxy-dihydroflavone | *Alkanna*, *Arnebia*, *Echium*, *Lithospermum*, *Onosma*,  *Glandora* | ^73, 78, 102-115^ |
|  |  |  | 5,4'-dihydroxy-7,3'-dimethoxydihydroflavones |  |  |
|  |  |  | 5,7,4'-trihydroxy-3,6,8-trimethoxyflavone |  |  |
|  |  |  | apigenin |  |  |
|  |  |  | apigenin-7-O-β-D glucopyranoside |  |  |
|  |  |  | chrysoeriol-7-O-β-D-glucopyranoside |  |  |
|  |  |  | cyanidin |  |  |
|  |  |  | cyanidin 3-glucoside |  |  |
|  |  |  | isorhamnetin-3-O-rutinoside |  |  |
|  |  |  | kaempferol-3-O-neohesperidoside |  |  |
|  |  |  | luteolin-7-glucoside |  |  |
|  |  |  | luteolin-7-O-β-D-glucopyranoside |  |  |
|  |  |  | myricetin |  |  |
|  |  |  | quercetin |  |  |
|  |  |  | rutin |  |  |
|  |  |  | delphinidin |  |  |
|  |  |  | glabrol |  |  |
|  |  |  | glabridin |  |  |
| Others | Alkanna |  | alkandiol | *Arnebia*, *Echium*, *Lithospermum*, *Lobostemon*,*Glandor,Alkanna* | ^48, 50, 59, 78, 94, 96, 116-126^ |
|  |  |  | 9,17-epoxyarnebinol |  |  |
|  |  |  | allantoin |  |  |
|  |  |  | arnebianoid |  |  |
|  |  |  | arnebia aldehyde |  |  |
|  |  |  | arnebia naphthalenol |  |  |
|  |  |  | cis-β-Ocimene |  |  |
|  |  |  | clavilactone A |  |  |
|  |  |  | cumene |  |  |
|  |  |  | daucosterol |  |  |
|  |  |  | epoxyarnebinol |  |  |
|  |  |  | uracil |  |  |
|  |  |  | urea |  |  |
|  |  |  | β-Cedrene |  |  |
|  |  |  | β-Copaene |  |  |
|  |  |  | β-Cubebene |  |  |
|  |  |  | β-sitosterol |  |  |
|  |  |  | α-Pinene |  |  |
|  |  |  | α-Selinene |  |  |
|  |  |  | mifepristone |  |  |
|  |  |  | osthol |  |  |
|  |  |  | oxymorphone |  |  |
|  |  |  | pinoresinol |  |  |
|  |  |  | β-Bourbonene |  |  |
|  |  |  | jolkinolide E |  |  |
|  |  |  | 2-(2Z)-(3-hydroxy-3,7-di  methylocta-2,6-dienyl)-1,  4-benzenediol |  |  |
|  |  |  | 1,8-Cineole |  |  |

**References**

1. Aga, E., Nie, L., Dong, Z. Z., Wang, J. Multi-component quantitative analysis combined with chromatographic fingerprint for quality assessment of Onosma hookeri. *China Journal of Chinese Materia Medica* **40**(22), 4442-4445.(2015)

2. An, S., Park, Y. D., Paik, Y. K., Jeong, T. S., Lee, W. S. Human ACAT inhibitory effects of shikonin derivatives from Lithospermum erythrorhizon. *Bioorganic & Medicinal Chemistry Letters* **17**(4), 1112-1116. (2007)

3. Assimopoulou, A. N., Boskou, D., Papageorgiou, V. P. Antioxidant activities of alkannin, shikonin and Alkanna tinctoria root extracts in oil substrates. *Food Chemistry* **87**(3), 433-438. (2004)

4. Assimopoulou, A. N., Papageorgiou, V. P. Radical scavenging activity of Alkanna tinctoria root extracts and their main constituents, hydroxynaphthoquinones. *Phytotherapy Research* **19**(2), 141–147. (2005)

5. Bai, X. R., Zhang, N., Ren, K., Xu, J. P., Li, Z. Y., Zhang, C. H, et al. Chemical constituents from Stenosolenium saxatile (Pall.) Turcz. (Boraginaceae). *Biochemical Systematics & Ecology*  **74**, 30-32. (2017)

6. Brockmann, H. Die Konstitution des Alkannins, Shikonins und Alkannans. *European Journal of Organic Chemistry* **521**(1), 1-47. (2010)

7. Chaouche, T. M., Haddouchi, F., Bekkara, F. A. Identification of shikonin from the roots of Echium pycnanthum Pomel. *Asian Journal of Pharmaceutical and Clinical Research* **5**(3), 30-32. (2012)

8. Cho, M. H., Paik, Y. S., Hahn, T. R. Propionylshikonin from the roots of Lithospermum erythrorhizon. *Archives of Pharmacal Research* **22**(4), 414-416. (1999)

9. Choi., Yung., Hyun., Kim., Gi-Young., Prasad. Shikonin Isolated from Lithospermum erythrorhizon Downregulates Proinflammatory Mediators in Lipopolysaccharide-Stimulated BV2 Microglial Cells by Suppressing Crosstalk between Reactive Oxygen Species and NF-kappa B. *Biomolecules & Therapeutics*. (2015)

10. Fujita., Hara., Ogino., Suga. Production of shikonin derivatives by cell suspension cultures of Lithospermum erythrorhizon : I. Effects of nitrogen sources on the production of shikonin derivatives. *Plant Cell Reports*. (1981)

11. Huang, Z. A., Zhang, M. B., Ma, L. A., Gu, L. A survey of chemical and pharmacologic studies on zicao (Arnebia sp., Lithospermum sp.,and Onosma sp.). *Natural Product Research & Development*. (2000)

12. Ko, F. N., Lee, Y. S., Kuo, S. C., Chang, Y. S., Teng, C. M. Inhibition on platelet activation by shikonin derivatives isolated from Arnebia euchroma. *Biochimica et Biophysica Acta (BBA) - Molecular Cell Research* **1268**(3), 329-334. (1995)

13. Lattoo, S. K., Koul, S., Dhar, M. K., Khajuria, R. K., Gupta, D. K., Dhar, A. K. Production of β-β,Dimethylacrylshikonin in Callus Cultures ofOnosmaechioides VarhispidumClarke. *Journal of Plant Biochemistry & Biotechnology* **14**(2), 193-195. (2005)

14. Nikita, G., Vivek, P., Chhaya, G. Wound-healing activity of an oligomer of alkannin/shikonin, isolated from root bark of Onosma echioides. *Natural Product Research* **1**. (2015)

15. Ozgen, U., Ozturk, M., Atila, A., Sevindik, H. G., Coskun, M. Quantitative HPLC Analysis of Deoxyshikonin, Acetyl shikonin and 3-Hydroxy-isovaleryl shikonin in Onosma armeniacum root. *Planta Medica* **73**(9), 883. (2007)

16. Pietrosiuk, A., Skopińska-Różewska, E., Furmanowa, M., Wiedenfeld, H., Sommer, E., Sokolnicka, I. Immunomodulatory effect of shikonin derivatives isolated from Lithospermum canescens on cellular and humoral immunity in Balb/c mice. *Pharmazie Die*.(2004)

17. Romanova, A. S., Tareeva, N. V., Ban'Kovskii, A. I. Isolation of shikonin from Onosma caucasicum and Echium rubrum. *Chemistry of Natural Compounds* **3**(1), 60-60.(1967)

18. Sharma, N., Sharma, U. K, Malik, S., Bhushan, S., Kumar, V., Verma, S. C. Isolation and purification of acetylshikonin andb-acetoxyisovalerylshikonin from cell suspensioncultures of Arnebia euchroma (Royle) Johnstonusing rapid preparative HPLC. **31**(4), 629-635.(2008)

19. Shcherbanovskii, L. R., Luks, Y. A. Shikonin from Echium lycopsis. *Chemistry of Natural Compounds* **10**(4), 517-517. (1974)

20. Shukla, Y. N, Tandon, J. S., Bhakuni, D. S., Dhar, M. M. Naphthaquinones of Arnebia nobilis. *Phytochemistry* **10**(8), 1909-1915.(1971)

21. Skoneczny, D., Zhu, X., Weston, P. A., Gurr, G. M., Callaway, R. M., Weston, L. A. Production of pyrrolizidine alkaloids and shikonins in Echium plantagineum L. in response to various plant stressors. *Pest Management Science* **75**(9).(2019)

22. Sut, S., Pavela, R., Kolar?Ik, V., Lupidi, G., Maggi, F., Dall’Acqua S. Isobutyrylshikonin and isovalerylshikonin from the roots of Onosma visianii inhibit larval growth of the tobacco cutworm Spodoptera littoralis. *Industrial Crops and Products* 109, 266-273.(2017)

23. Toker, A., Akcay, F., Aksoy, H., Suleyman, H., Erdem, H. The effects of acetyl shikonin isolated from Onosma armeniacum on oxidative stress in ethanol-induced ulcer model of rats. *Turkish Journal of Medical Sciences* **43**(2), 315-320.(2013)

24. Yasuhiro., Fujita., Yasuhiro., Hara., Chuzo., SugaTeijiro. Production of shikonin derivatives by cell suspension cultures of Lithospermum erythrorhizon. *Plant Cell Reports*.(1981)

25. Yazaki, K., Matsuoka, H., Ujihara, T., Sato, F. Shikonin Biosynthesis in Lithospermum erythrorhizon. (2010)

26. Assimopoulou, A. N., Ganzera, M., Stuppner, H., Papageorgiou, V. P. Simultaneous determination of monomeric and oligomeric alkannins and shikonins by high-performance liquid chromatography-diode array detection-mass spectrometry. *Biomedical Chromatography* **22**(2), 173-190.(2010)

27. Dong, M., Liu, D., Li, Y. H., Chen, X. Q., Li, R. T. Naphthoquinones from Onosma paniculatum with Potential Anti-inflammatory Activity. *Planta Medica* **83**(7), 631-635.(2017)

28. Yang, Y., Zhao, D., Yuan, K., Zhou, G., Wang, Y., Xiao, Y. Two new dimeric naphthoquinones with neuraminidase inhibitory activity from Lithospermum erythrorhizon. *Natural Product Research* **29**(10), 908-913.(2015)

29. Ye, J. Study on chemical constituents and antibacterial activity of Lithospermum erythorhizon. *Fudan University*. (2009).

30. Ahmad, L., Yi, H., Hao, J. C., Semotiuk, A., Liu, Q. R., Mazari, P. Toxic pyrrolizidine alkaloids provide a warning sign to overuse of the ethnomedicine Arnebia benthamii. *Journal of Ethnopharmacology* 88-94.(2017)

31. Chung, B. Y., Lee, Y. B., Baek, M. H, Kim, J. H., Wi, S. G., Kim, J. S. Effects of low-dose gamma-irradiation on production of shikonin derivatives in callus cultures of Lithospermum erythrorhizon S. *Radiation Physics & Chemistry* **75**(9), 1018-1023.(2006)

32. Hiroyuki., Inouye., Haruki., Matsumura. Two quinones from callus cultures of Echium lycopsis. *Phytochemistry* **20**(7), 1701-1705.(1981)

33. Papageorgiou, V., Digenis, G. Isolation of two New Alkannin Esters from Alkanna tinctoria. *Planta Medica* **39**(05), 81-84.(1980)

34. Rajbhandari, M., Schoepke, T. H., Mentel, R., Lindequist, U. Antibacterial and antiviral naphthazarins from Maharanga bicolor. *Pharmazie* **62**(8), 633-635.(2007)

35. Sayyada, K., Mehrotra, S. Pharmacognostical study of Japanese drug 'Nan-Shikon' root of Arnebia euchsoma (Royle). Johnston growing in India. *Natural Science* **51**(4), 171-177.( 2000)

36. Sevimli-Gur, C., Akgun, I. H., Deliloglu-Gurhan, I., Korkmaz, K. S, Bedir, E. Cytotoxic naphthoquinones from Alkanna cappadocica ( perpendicular). *Journal of Natural Products* **73**(5).(2010)

37. Tepe, M., Atilla, D., erer, V. S., Ifti, Y. O., Ek, Er. M. G. Effects of Chemical Stimulations on Shikonin and Derivatives in In Vitro Turkish Echium italicum L. Hairy Root Culture. (2019)

38. Tung, N. H., Du, G. J., Wang, C. Z., Yuan, C. S., Shoyama, Y. Naphthoquinone Components from Alkanna tinctoria (L.) Tausch Show Significant Antiproliferative Effects on Human Colorectal Cancer Cells. *Phytotherapy Research* **27**(1).(2013)

39. Türkiye, Y., Apos., de., Yetien, B., Echium, T., Ikonin, T. A simple isocratic high-perfomance liquid chromatography method for the simultaneous determination of shikonin derivatives in some echium species growing wild in turkey. (2018)

40. Feng, W. W., Li, G. Y., Tan, Y., Wang, H. Y., & Wang, J. H. Studies on the chemical constituents of naphthoquinones from Arnebia euchroma and Lithospermum erythorhizon. *Modern Chinese Medicine.* **12**, 4. (2010).

41. Hu, J., & Pu, Q. H. Studies on the chemical constituents of Onosma paniculatum. Yunnan Journal of Traditional Chinese Medicine. **29**, 29-29. (2008)

42. Li, F., Li, Y. H., Li, J. H., Li, R. T. Studies on the chemical constituents of Onosma paniculatum. *Yunnan Journal of Traditional Chinese Medicine.* **35**, 70-70. (2014).

43. Graikou, K., Baranek, K. S., Pietrosiuk, A., Damianakos, H., Jeziorek, M., Chinou, I. Chemical analysis of Lithospermum canescens (Michx.) Lehm. hairy roots through TLC, CC, GC-MS, ESI-MS and NMR methods. *Acta Chromatographica* **23**(2), 353-363.(2011)

44. Liao, M., Jiang, H. L., Medicine, S. O., University, J. Simultaneous Determination of 13 Shikonins and Shikonofurans in Medicinal Arnebia plants by HPLC-MS. *Chinese Pharmaceutical Journal*.(2016)

45. Sun, B., Jiang, H., Wang, Z. N., Luo, H. Z., Jia, A. Q. Phytochemical constituents of Onosma bracteatum Wall. *Phytochemistry Letters* **45**(6), 1-5.(2021)

46. Yoshizaki, F., Hisamichi, S., Kondo, Y., Sato, Y., Nozoe, S. Studies on Shikon. III. New Furylhydroquinone Derivatives, Shikonofurans A, B, C, D and E, from Lithospermum erythrorhizon SIEB. et ZUCC. *Chemical & Pharmaceutical Bulletin* **30**(12), 4407-4411.(2008)

47. Fukui, H., Tsukada, M., Mizukami, H., Tabata, M. Formation of stereoisomeric mixtures of naphthoquinone derivatives in Echium lycopsis callus cultures. *Phytochemistry* **22**(2), 453-456.(1983)

48. Li, H. M., Tang, Y. L., Zhang, Z. H., Liu, C. J., Xia, X. S. Compounds from Arnebia euchroma and Their Related Anti-HCV and Antibacterial Activities. *Planta Medica* **78**(1), 39-45.(2011)

49. Özgen, U., Coşkun, M., Kazaz, C., Seçen, H. Naphthoquinones from the roots of Onosma argentatum Hub.-Mor. (Boraginaceae). *Turkish Journal of Chemistry* **28**(4), 451-454.(2004)

50. Yao, X. S., Ebizuka, Y., Noguchi, H., Kiuchi, F., Shibuya, M., Iitaka, Y. Biologically active constituents of Arnebia euchroma: structures of new monoterpenylbenzoquinones: arnebinone and arnebifuranone. *Chemical & Pharmaceutical Bulletin* **39**(11), 2962-2964.(1991)

51. A., El-Shazly, T., Sarg, A., Ateya. Pyrrolizidine Alkaloids from Echium setosum and Echium vulgare. *Journal of Natural Products* **59**(3), 310–313.(1996)

52. Ahmad, L., He, Y., Semotiuk, A. J, Liu, Q. R., Hao, J. C. Survey of pyrrolizidine alkaloids in the tribe Lithospermeae (Boraginaceae) from Pan-Himalaya and their chemotaxonomic significance. *Biochemical Systematics and Ecology* **81**, 49-57.(2018)

53. Al-Snafi, AE. Chemical Constituents and Pharmacological Effects of Lithospermum officinale. (2019)

54. Betteridge, K., Cao, Y. S. Improved method for extraction and LC-MS analysis of pyrrolizidine alkaloids and their N-oxides in honey: application to Echium vulgare honeys. *Journal of Agricultural & Food Chemistry* **53**(6), 1894-1902.(2005)

55. Biondi, M., D'Alessandro, P. Taxonomical revision of the Longitarsus capensis species-group: An example of Mediterranean-southern African disjunct distributions (Coleoptera: Chrysomelidae). *European Journal of Entomology* **105**, 719-736.(2008)

56. Boppré, M., Colegate, S. M., Edgar, J. A. Pyrrolizidine alkaloids of Echium vulgare honey found in pure pollen. *J Agric Food Chem* **53**(3), 594-600.(2005)

57. Da, Mianakos, H., Sotiroudis. G., Chinou, I. Pyrrolizidine alkaloids from Onosma erecta. *Journal of Natural Products* **76**(10), 1829.(2013)

58. El-Shazly, A., Abdel-Ghani, A., Wink, M. Pyrrolizidine alkaloids from Onosma arenaria (Boraginaceae). *Biochemical Systematics & Ecology* **31**(5), 477-485.(2003)

59. Hamid, A., Khan., Indrani., Chandrasekharan. Naphthazarins from Arnebia hispidissima. *Phytochemistry* **22**(2), 614-615.(1983)

60. Heide, L., Rengel, B., R?Der E., Tabata, M. Absence of Toxic Pyrrolizidine Alkaloids in Cultured Cells of <EM EMTYPE=. *Planta Medica* **55**(7), 684-684.(1989)

61. Krenn, L., Wiedenfeld, H., Roeder, E. Pyrrolizidine alkaloids from Lithospermum officinale. *Phytochemistry* **37**(1), 275-277.(1994)

62. Maria, M., Romeiras, A. B. D., Lia, Ascenso, C., Maria, C., Duarte, B., Maria, A., Diniz, B., A, MSP. Taxonomy of Echium (Boraginaceae) species from Cape Verde Islands. *Australian Systematic Botany* **21**(1), 26-38. (2008)

63. Mroczek, T., Ndjoko, K., G?Owniak, K., Hostettmann, K. On-line structure characterization of pyrrolizidine alkaloids in Onosma stellulatum and Emilia coccinea by liquid chromatography-ion-trap mass spectrometry. *Journal of Chromatography A* **1056**(1-2), 91-97.(2004)

64. Ourania., Kretsi., Nektarios., Aligiannis., AlexiosLeandros., Skaltsounis. Pyrrolizidine Alkaloids from Onosma leptantha. *Helvetica Chimica Acta* **86**(9), 3136-3140.(2003)

65. Pietrosiuk, A., Sykłowska-Baranek, K., Wiedenfeld, H., Wolinowska, R., Furmanowa, M., Jaroszyk, E. The shikonin derivatives and pyrrolizidine alkaloids in hairy root cultures of Lithospermum canescens (Michx.) Lehm. *Plant Cell Reports* **25**(10), 1052-1058.(2006)

66. R?Der, E., Rengel-Mayer, B. Pyrrolizidine Alkaloids from Arnebia euchroma*. *Planta Medica* **59**(2).(1993)

67. RÖDER, E,, Wiedenfeld, H., Kroger, R. Pyrrolizidinalkaloide dreier Onosma-Sippen (Bora- gina cea e-Lithospermeae).

68. Roeder, E., Rengel, B. Pyrrolizidine alkaloids from Lithospermum erythrorhizon. *Phytochemistry* **29**(2), 690-693.(1990)

69. Roeder, E., Wiedenfeld, H., Schraut, R. Pyrrolizidine alkaloids from Alkanna tinctoria. *Phytochemistry* **23**(9), 2125-2126.(1984)

70. Smyrska, W., Natalia., Mroczek., Tomasz., Wojtanowski., Krzysztof. Comparative HILIC/ESI-QTOF-MS and HPTLC studies of pyrrolizidine alkaloids in flowers of Tussilago farfara and roots of Arnebia euchroma. *Phytochemistry Letters* **20**, 339-349. (2017)

71. Smyrska-Wieleba, N., Wojtanowski, K. K., Mroczek, T. Comparative HILIC/ESI-QTOF-MS and HPTLC studies of pyrrolizidine alkaloids in flowers of Tussilago farfara and roots of Arnebia euchroma. *Phytochemistry Letters* 339-349. (2016)

72. Wiedenfeld, H., Pietrosiuk, A., Furmanowa, M., Roeder, E. Pyrrolizidine Alkaloids from Lithospermum canescens Lehm. *Zeitschrift Für Naturforschung C* **58**(3-4). (2003)

73. Ff, A., Pba, A., Ff, B., Gi, B., Spc, D., Pv, A. The chemical composition on fingerprint of Glandora diffusa and its biological properties. *Arabian Journal of Chemistry* **10**(5), 583-595. (2017)

74. Kashiwada, Y., Nishizawa, M., Yamagishi, T., Tanaka, T., Nonaka, G. I., Cosentino, L. M. Anti-AIDS agents, 18. Sodium and potassium salts of caffeic acid tetramers from Arnebia euchroma as anti-HIV agents. *Journal of Natural Products* **58**(3), 392-400. (1995)

75. Qin, D. M., He, J. H., & Rena, K. S. M. Study on water-soluble chemical constituents of Arnebia euchroma. *Journal of Xinjiang Medical University*. **3**. (2009)

76. A, H.Y, A, K. I, B, K Y. Caffeic acid oligomers in Lithospermum erythrorhizon cell suspension cultures. *Phytochemistry* **53**(6), 651-657. (2000)

77. Ferreres, F., Vinholes, J., Gil-Izquierdo, A., Valent?O, P., Gon?Alves, R. F., Andrade, P. B. In vitro studies of α-glucosidase inhibitors and antiradical constituents of Glandora diffusa (Lag.) D.C. Thomas infusion. *Food Chemistry* **136**(3-4), 1390-1398.(2013)

78. Harnett, S. M., Oosthuizen, V., Venter, M. Anti-HIV activities of organic and aqueous extracts of Sutherlandia frutescens and Lobostemon trigonus. *Journal of Ethnopharmacology* **96**(1-2), 113-119.(2005)

79. Yamamoto, H., Yazaki, K., Inoue, K. Simultaneous analysis of shikimate-derived secondary metabolites in Lithospermum erythrorhizon cell suspension cultures by high-performance liquid chromatography. *Journal of Chromatography B Biomedical Sciences & Applications* **738**(1), 3-15.(2000)

80. Chawuke, P., Berg, N., Fouche, G., Maharaj, V., Alexandre, K. B. Lobostemon trigonus (Thunb.) H. Buek, a medicinal plant from South Africa as a potential natural microbicide against HIV-1. *Journal of Ethnopharmacology* **277**(1), 114222.(2021)

81. Kirkan, B., Sarikurkcu, C., Ozer, M. S., Cengiz, M., Atılgan, N., Ceylan, O. Phenolic profile, antioxidant and enzyme inhibitory potential of Onosma tauricum var. tauricum. *Industrial Crops and Products* 125, 549-555.(2018)

82. Mitra, M., Mohammadreza, S. A., Alireza, G., Dehkordi, N. G., Jazi, S. Production of Rosmarinic Acid in Echium amoenum Fisch. and C.A. Mey. Cell Cultures. *iranian journal of pharmaceutical research*. (2005)

83. Mizukami, H., Ogawa, T., Ellis, O. Induction of rosmarinic acid biosynthesis in Lithospermum erythrorhizon cell suspension cultures by yeast extract. *Plant Cell Reports*.(1992)

84. Tufa, T,, Damianakos, H., Zengin, G., Graikou, K., Chinou, I. Antioxidant and enzyme inhibitory activities of disodium rabdosiin isolated from Alkanna sfikasiana Tan, Vold and Strid. *South African Journal of Botany* S0254629917316988. (2018)

85. Yamamura, Y,, Ogihara, Y., Mizukami, H. Cinnamic acid 4-hydroxylase from Lithospermum erythrorhizon: cDNA cloning and gene expression. *Plant Cell Reports* **20**(7), 655-662.(2001)

86. Yilanci, S., Bali, Y. Y, Yuzbasioglu, M., Unlu, R. E, Kuruuzum-Uz, A. The evaluation of wound healing potential of rosmarinic acid isolated from Arnebia purpurea. In: *Planta Medica* (2015).

87. A, S. N., B, S. A., B, S. A. R., A, S. A. S., A, R. S. Antibacterial activity directed isolation of compounds from Onosma hispidum. *Microbiological Research* **161**(1), 43-48.(2006)

88. Arumugam, R., Sarikurkcu, C., Ozer, M. S. Comparison of methanolic extracts of Doronicum orientale and Echium angustifolium in terms of chemical composition and antioxidant activities. *Biocatalysis and Agricultural Biotechnology* **33**, 101984.(2021)

89. Kelley, C. J., Harruff, R. C., Carmack, M. Polyphenolic acids of Lithospermum ruderale. II. Carbon-13 nuclear magnetic resonance of lithospermic and rosmarinic acids. *Journal of Organic Chemistry* **41**(3), 449-455.(1976)

90. Liao, M., Yuan, Y., Feng, C., Zhang, Y. Studies on Chemical Constituents from Arnebia guttata Bunge. *Journal of South-Central University for Nationalities(Natural Science Edition)*. (2018)

91. Lodama, K. E., Toit, E., Steyn, J. M., Araya, H. T., Plooy, C. Breaking seed dormancy in Lobostemon fruticosus. *Acta Horticulturae* **1204**,115-122. (2018)

92. Sarikurkcu, C., Sahinler, S. S., Tepe, B. Onosma aucheriana, O. frutescens, and O. sericea: Phytochemical profiling and biological activity. *Industrial Crops and Products* **154**. (2020)

93. Yazaki K, Heide L, Tabata M. Formation of p-hydroxybenzoic acid from p-coumaric acid by cell free extract of Lithospermum erythrorhizon cell cultures. *Planta Medica* **56**(7), 616-616.(1990)

94. Yuzbasioglu, Kuruuzum-Uz, Guvenalp, Simon, Toth, Harput, et al. Cytotoxic Compounds from Endemic Arnebia purpurea. *NAT PROD COMMUN* **10**(4), 595-596. (2015)

95. Zannou, O., Pashazadeh, H., Ghellam, M., Koca, I., Kaddour, A. A., Leriche, F. Appraisal of phenolic compounds, antioxidant activity and in vitro gastrointestinal digestion of borage (Echium amoenum) flowers using natural deep eutectic solvent (NADES). (2022)

96. Huang, Y. Study on chemical constituents and antibacterial activity of Onosma hookeri. *Southwest Jiaotong University*. (2012)

97. Alhazzaa, R., Bridle, A. R, Mori, T. A., Barden, A. E., Nichols, P. D., Carter, C. G. Echium oil is better than rapeseed oil in improving the response of barramundi to a disease challenge. *Food Chemistry* **141**(2), 1424-1432. (2013)

98. Bell, J. G., Strachan, F., Good, J. E., Tocher, D. R. Effect of dietary echium oil on growth, fatty acid composition and metabolism, gill prostaglandin production and macrophage activity in Atlantic cod (Gadus morhua L.). *Aquaculture Research* **37**. (2010)

99. Gómez-Mercado, C. Occurrence and characterization of oils rich in γ-linolenic acid Part II: fatty acids and squalene from Macaronesian Echium leaves. *Phytochemistry*. (2000)

100. Guil-Guerrero, J. L., Gómez-Mercado, F., Garca-Maroto, F. Occurrence and characterization of oils rich in γ-linolenic acid. Part I : Echium seeds from Macaronesia. *Phytochemistry* **54**(4), 525-529. (2000)

101. López-Martínez, J., Campra-Madrid, P., Guil-Guerrero, J. L. γ-Linolenic Acid Enrichment from Borago officinalis and Echium fastuosum Seed Oils and Fatty Acids by Low Temperature Crystallization. *Journal of Bioscience & Bioengineering* **97**(5), 294-298. (2004)

102. Al, NSME. Invitro antioxidant and free radical scavenging activity of Leonurus cardiaca subsp. Persicus ,Grammosciadium platycarpum and Onosma demawendicum. *African Journal of Biotechnology* **9**, 8865-8871. (2016)

103. Antonios, S., Mellidis, Vassilios, P., Papageorgiou. Phenolic Constituents from Onosma heterophylla. *Journal of Natural Products* **56**(6), 949-952. (1993)

104. Bame, J., Graf, T., Junio, H., Bussey, R., Jarmusch, S., El-Elimat, T. Sarothrin from Alkanna orientalis Is an Antimicrobial Agent and Efflux Pump Inhibitor. *Planta Medica* **79**(05), 327-329. (2013)

105. Botelho, J., Gaspar, L., Gonaves, R. F., Valento, P., Andrade, P, B. Metabolic profiling and bioactivity of Lithospermum diffusum. In: *IJUP'11.* (2011)

106. El-Mawla, A. Effect of certain elicitors on production of pyrrolizidine alkaloids in hairy root cultures of Echium rauwolfii. *Die Pharmazie* **65**(3), 224. (2010)

107. Esfahani, R. M., Salimikia, I., Yazdinezhad, A. R., Golfakhrabadi, F. In vitro antioxidant and free radical scavenging activity of four Alkanna species growing in Iran. *Pharmacognosy Research* **7**(1). (2015)

108. Fan, W. J., Li, Y. X., Lu, H. M,, Xu, F,, Liu, G. B. Study on mechanism of Radix astragali-Lithospermum erythrorhizon in treatment of diabetic ulcer based on network pharmacology. *Journal of Hainan Medical College* **27**(8):6. (2021)

109. Ferreres, F., Pereira, D. M., Valent?O, P., Andrade, P. B. First report of non-coloured flavonoids in Echium plantagineum bee pollen: differentiation of isomers by liquid chromatography/ion trap mass spectrometry. *Rapid Commun Mass Spectrom* **24**(6), 801-806. (2010)

110. Kelley, C. J., Mahajan, J. R., Brooks, L. C., Neubert, L. A., Breneman, W. R., Carmack, M. Polyphenolic acids of Lithospermum ruderale (Boraginaceae). I. Isolation and structure determination of lithospermic acid. *Chemischer Informationsdienst* **6**(40). (1975)

111. Kelley, C. J., Mahajan, J. R., Brooks, L. C., Neubert. L. A., Breneman, W. R., Carmack, M. ChemInform Abstract: POLYPHENOLIC ACIDS OF LITHOSPERMUM RUDERALE DOUGL. EX LEHM, (BORAGINACEAE) PART 1, ISOLATION AND STRUCTURE DETERMINATION OF LITHOSPERMIC ACID. *Chemischer Informationsdienst* **6**. (1975)

112. Lu, L., Shu, C., Chen, L., Wang, C., Zhou, E. The impacts of natural antioxidants on sclerotial differentiation and development in Rhizoctonia solani AG-1 IA. *European Journal of Plant Pathology* **146**(4), 729-740. (2016)

113. Wang, X., Tang, Z. S., Yang, N., Yue, Z. G. Protein Tyrosine Phosphatase 1B(PTP1B) Inhibitors from Arnebia euchroma. *Chinese Pharmaceutical Journal*. (2016)

114. Wyk B, Winter P, Buys MH. The Major Flower Anthocyanins of Lobostemon (Boraginaceae). *Biochemical Systematics & Ecology* **25**(1), 39-42. (1997)

115. Zengin, G., Ceylan, R., Katani? J., Aktumsek, A., Mati? S., Boroja, T. Exploring the therapeutic potential and phenolic composition of two Turkish ethnomedicinal plants – Ajuga orientalis L. and Arnebia densiflora (Nordm.) Ledeb. *Industrial Crops and Products* 116, 240-248. (2018)

116. Cao, H., Zhang, W., Liu, D., Hou, M., Shao, M. Identification, in vitro evaluation and modeling studies of the constituents from the roots of Arnebia euchroma for antitumor activity and STAT3 inhibition. *Bioorganic Chemistry* **96**, 103655. (2020)

117. El-Rokh, A. R., Negm, A., El-Shamy, M., El-Gindy, M., Abdel-Mogib, M. Insecticidal Activity of Nitraria retusa and Echium angustifolium Extracts and Active Metabolites against Aphis craccivora and Bemicia tabaci. (2019)

118. Han, J., Weng, X., Bi, K. Antioxidants from a Chinese medicinal herb - Lithospermum erythrorhizon. *Food Chemistry* **106**(1), 2-10. (2008)

119. Jie, H,, Weng, X,, Bi, K. Antioxidants from a Chinese medicinal herb – Lithospermum erythrorhizon. *Food Chemistry* **106**(1), 2-10. (2008)

120. Paola, P., Manuela, L., Monica, N., Maurizio, B., Antonella M, Sergio R, et al. Chemical composition, in vitro antitumor and pro-oxidant activities of Glandora rosmarinifolia (Boraginaceae) essential oil. *Plos One* **13**(5), e0196947-.(2018)

121. Park, J. Y., Lee, S., Han, S., Kim, H. M., Lee, J. M., Lee, S. A Paraben Derivative from the Seeds of Lithospermum erythrorhizon. *Journal of the Korean Society for Applied Biological Chemistry* **52**(6), 643-645. (2009)

122. Park, J. Y., Lee, S. L., Han, S., Kim, H. M., Lee, J. M, Ahn, Y. H. Phytochemical Constituents from the Seeds of Lithospermum erythrorhizon. *Natural Product Sciences* **15**(4), 181-184. (2009)

123. Santana, O., Reina, M., Fraga, B. M., Sanz, J., González-Coloma, A. Antifeedant activity of fatty acid esters and phytosterols from Echium wildpretii. *Chemistry & Biodiversity* **9**(3), 567-576. (2012)

124. Xin, G. X., Wang, B. Z., Sun, Z. R., Zhang, J. W., Zhang, H. G. Chemical Constituents from Root of Arnebia euchroma(Royle)Johnst. *Journal of Jilin University* 48(2), 319-322. (2010)

125. Zcan., Süzerer, J., Mater. Variation of some seed oil components at altitidunal range in a widely distributed species, Echium italicum L. (Boraginaceae) from Turkey. (2020)

126. Zhang, X. T., Yin, Z. Q., Ye, W. C., Ni, L., Zhao, S. X. Chemical Constituents from Lithospermum zollingeri. *Chinese Journal of Natural Medicines* **3**(6), 357-358. (2005)
